# Genome-Wide and Differential Proteomic Analysis of Hepatitis B Virus and Aflatoxin B1 Related Hepatocellular Carcinoma in Guangxi, China

**DOI:** 10.1371/journal.pone.0083465

**Published:** 2013-12-31

**Authors:** Lu-Nan Qi, Le-Qun Li, Yuan-Yuan Chen, Zhao-Hong Chen, Tao Bai, Bang-De Xiang, Xiao Qin, Kai-Yin Xiao, Min-Hao Peng, Zhi-Ming Liu, Tang-Wei Liu, Xue Qin, Shan Li, Ze-Guang Han, Zeng-Nan Mo, Regina M. Santella, Cheryl A. Winkler, Stephen J. O’Brien, Tao Peng

**Affiliations:** 1 Department of Hepatobiliary Surgery, Tumor Hospital of Guangxi Medical University, Nanning, Guangxi Province, China; 2 Department of Ultrasound, First Affiliated Hospital of Guangxi Medical University, Nanning, Guangxi Province, China; 3 Department of Hepatobiliary Surgery, First Affiliated Hospital of Guangxi Medical University, Nanning, Guangxi Province, China; 4 Department of Clinical Laboratory, First Affiliated Hospital of Guangxi Medical University, Nanning, Guangxi Province, China; 5 China National Human Genome Center at Shanghai, Shanghai, China; 6 Department of Urology and Nephrology Surgery, First Affiliated Hospital of Guangxi Medical University, Nanning, Guangxi Province, China; 7 Department of Environmental Health Sciences, Mailman School of Public Health, Columbia University, New York, New York, United States of America; 8 Laboratory of Genomic Diversity, National Cancer Institute, NIH, Frederick, Maryland, United States of America; The University of Hong Kong, China

## Abstract

Both hepatitis B virus (HBV) and aflatoxin B1 (AFB1) exposure can cause liver damage as well as increase the probability of hepatocellular carcinoma (HCC). To investigate the underlying genetic changes that may influence development of HCC associated with HBV infection and AFB1 exposure, HCC patients were subdivided into 4 groups depending upon HBV and AFB1 exposure status: (HBV(+)/AFB1(+), HBV(+)/AFB1(-), HBV(-)/AFB1(+), HBV(-)/AFB1(-)). Genetic abnormalities and protein expression profiles were analyzed by array-based comparative genomic hybridization and isobaric tagging for quantitation. A total of 573 chromosomal aberrations (CNAs) including 184 increased and 389 decreased were detected in our study population. Twenty-five recurrently altered regions (RARs; chromosomal alterations observed in ≥10 patients) in chromosomes were identified. Loss of 4q13.3-q35.2, 13q12.1-q21.2 and gain of 7q11.2-q35 were observed with a higher frequency in the HBV(+)/AFB1(+), HBV(+)/AFB1(-) and HBV(-)/AFB1(+) groups compared to the HBV(-)/AFB(-) group. Loss of 8p12-p23.2 was associated with high TNM stage tumors (*P* = 0.038) and was an unfavorable prognostic factor for tumor-free survival (*P* =0.045). A total of 133 differentially expressed proteins were identified in iTRAQ proteomics analysis, 69 (51.8%) of which mapped within identified RARs. The most common biological processes affected by HBV and AFB1 status in HCC tumorigenesis were detoxification and drug metabolism pathways, antigen processing and anti-apoptosis pathways. Expression of AKR1B10 was increased significantly in the HBV(+)/AFB1(+) and HBV(-)/AFB1(+) groups. A significant correlation between the expression of AKR1B10 mRNA and protein levels as well as AKR1B10 copy number was observered, which suggest that AKR1B10 may play a role in AFB1-related hepatocarcinogenesis. In summary, a number of genetic and gene expression alterations were found to be associated with HBV and AFB1- related HCC. The possible synergistic effects of HBV and AFB1 in hepatocarcinogenesis warrant further investigations.

## Background

Hepatocellular carcinoma (HCC) is one of the most prevalent human cancers worldwide [1]. Epidemiological evidence suggests that several environmental factors are involved in the development of HCC [2,3]. In Japan and the United States, more than 70% of cases are related to chronic HCV infection [4,5], while in Southern China and sub-Saharan Africa, HCC is associated with high dietary exposure to aflatoxin B1 (AFB1) and hepatitis B virus (HBV) infection and is the major causes of cancer mortality in these geographic areas [3–5]. 

Hepatocarcinogenesis is a complex process associated with the accumulation of genetic abnormalities that occur during initiation, promotion, and progression of the disease [6]. Both HBV infection and AFB1 exposure can cause liver damage, and increase the probability of HCC [2,7]. They appear to play different roles in HCC development due to their various biological effects. AFB1 is the most prevalent and carcinogenic of the aflatoxins. When ingested, AFB1 is processed in the liver by the cytochromes P450 system to reactive epoxides which can damage DNA [8,9]. A number of studies have confirmed that more than 50% of HCC patients who have been exposed to AFB1 carry a mutation in codon 249 (AGG^Arg^→AGT^Ser^ or AGG^Arg^→AGC^Ser^) of the p53 gene [9–11]. This hotspot mutation is considered a molecular marker reflecting AFB1-induced DNA damage that eventually results in HCC [12–14]. 

Previous microarray chromosomal proteomic studies have identified genes or chromosomal regions that may be involved in hepatocarcinogenesis [15–20]. These findings indicate that development of HCC is a complex polygene and multi-pathway process [21]. Genetic pathways associated with development of HBV-related HCC include RB1, methylation of p16INK4a, and amplification of Cyclin D1 [21]. In contrast, AFB1 alters the protein sequence of the tumor suppressor p53 gene which regulates the cell cycle and is important in conserving genome stability. Other ways by which AFB1 may act to promote HCC are poorly understood. HBV and AFB1 are also thought to influence the activity of similar pathways to promote HCC. Currently, it is not clear if these two pathways can act synergistically to promote HCC via common or overlapping molecular mechanisms. It is important to understand how HBV and AFB1 affect HCC development in China where there is high exposure to both HBV and AFB1. 

In this study, we used array-based comparative genomic hybridization (aCGH) and Isobaric Tagging Reagent Quantitative (iTRAQ) proteomics to identify chromosomal regions and proteins/genes that are altered as a result of HBV and AFB1 exposure. The results of this study may provide additional markers and molecular targets for the diagnosis and treatment of these types of HCC.

## Methods

This study enrolled 157 patients with HCC from the Tumor Hospital of Guangxi Medical University and First Affiliated Hospital of Guangxi Medical University, Nanning, Guangxi Province, China. 32 of which were enrolled for array-based comparative genomic hybridization and isobaric tagging reagent quantitative analysis. The study was approved by the both hospitals’ institutional review boards and was performed according to the Declaration of Helsinki. All patients gave their informed consents.

### Study patients

All patients (ages 23-75 years) had HCC and were negative for HCV (as determined by serology and pathological analyses). All study patients were subjected to a rigorous screening procedure before they were categorized into subgroups. We defined HBV- related and AFB1- related status in this study by the following criteria: The HBV- related (HBV (+)) patients were defined as HBsAg-positive, HBeAb-positive (or HBeAg-positive) and anti-HBc-positive. These patients all had > 1000 copies of serum HBV-DNA. In contrast, the HBV-negative (HBV (-)) patients were negative for HBsAg, HBeAg and anti-HBc. They also had undetectable serum HBV-DNA. All HBV-negative (HBV (-)) patients were positive for HBsAb protective antibodies. AFB1-related patients were classified on the basis of a G to T transversion in codon 249 of the p53 gene (AGG^Arg^→AGT^Ser^ or AGG^Arg^→AGC^Ser^ ) that results in a change from Arg to Ser in the protein sequence. AFB1- related (AFB1(+)) status was defined as the presence of the codon 249 mutations in p53 together with positive staining for AFB1-DNA in HCC tissues (Figure **S1**). AFB1- negative (AFB1(-)) status was defined as the absence of mutations in codon 249 of p53 as well as absence of AFB1-DNA in HCC tissue. Specimens which were AFB1-DNA positive but the p53 codon 249 mutation negative or specimens which were the p53 codon 249 mutation positive but AFB1-DNA negative were excluded from the study. For the iTRAQ quantitative proteomics analysis, normal hepatic tissue from hepatic hemangioma, liver resections and liver transplant donors were used as normal controls.

### Immunohistochemical staining for AFB1-DNA[22]

Although the mutation in codon 249 of the p53 gene is associated with AFB1 intake, this mutation is not carcinogen-specific and may also be seen in patients with HBV-related or HCV-related HCC. Serum AFB1 albumin adducts have been shown to help determine AFB1 intake. However, in this study, we determined AFB1 intake using a modification of a previously described method for AFB1 exposed tissue AFB1-DNA adduct immunohistochemistry [22] . 

Briefly, 4-mm-thick paraffin sections were dewaxed in xylene and rehydrated using a descending alcohol gradient. The sections were then washed in 0.5 M glycine in phosphate-buffered saline (PBS; pH 7.2) to inhibit/remove fixing agents, which can cause nonspecific binding of immunoperoxidase or reduce 30, 30-diaminobenzidine tetrahydrochloride (DAB) levels. Endogenous peroxidase activity was blocked by incubating the sections in 0.3–0.5% H_2_O_2_ in absolute methanol for 30 min. The slides were then washed in Tris buffer for 5 min and incubated with a few drops of 3% normal goat serum for 30 min. The sections were incubated overnight at 4°C with a 1:100 dilution of anti-AFB1 antibody [monoclonal anti-aflatoxin B1 (6A10); Novus Biolocals Inc., Littleton, CO, USA]. After the sections were washed three times with PBS, they were incubated with a biotin-conjugated secondary antibody (Sigma-Aldrich; St. Louis, MO, USA) at 37°C with a 1:2000 dilution for 30-50 min, followed by peroxidase-conjugated streptavidin for 30 min. Peroxidase activity was detected by incubating the sections in DAB for 10 min followed by counterstaining with Mayer’s haematoxylin. The sections were then dehydrated in alcohol, cleared in xylene and mounted with malinol. The labeling index was used to evaluate sections immuno-stained for AFB1. All evaluations were independently performed by two pathologists (S.J. O’Brien and Z-N Mo) who were blinded to the patients’ status. At least 1000 nuclei were counted at random in noncancerous hepatocytes from at least two sections at a magnification of ×400. Sections in which ≥ 5% of the hepatic nuclei expressed AFB1 were considered to be AFB1 positive.

### Array-based comparative genomic hybridization (aCGH)

Genomic DNA from tumors and control lymphocytes was digested with AluI and RsaI (Promega, Madison, WI, US) and labeled with Cy5 and Cy3 respectively, using Agilent Genomic DNA Labeling Kit PLUS (Agilent Technologies, Santa Clara, CA, US) per manufacturers’ instructions. Labeled DNA fragments were purified and specific activity determined.

Array hybridization was performed using Agilent Oligo aCGH Hybridization Kit (Agilent Technologies) per manufacturer’s instructions and arrays were scanned by Agilent Microarray Scanner (Agilent Technologies). The Z-score for each chromosomal aberrations region was calculated using DNA Analytics 6.5.0.58 software (Agilent Technologies)

### DNA purification and analysis of exon 7 of p53 mutation

Tumor tissues and normal lymphocytes (control) were collected during liver resection and frozen at -80°C. All sample had > 70% viable tumor cells as determined by pathological examination. Genomic DNA was extracted from tumor samples and lymphocytes using the Dneasy Tissue kit (Qiagen, Hilden Germany) according to the manufacturer’s instructions. Exon 7 of p53 was amplified using the forward and reverse primers 5’-cttgccacaggtctccccaa-3’ and 5’-aggggtcagcggcaagcaga-3’ (237bp), respectively, under standard cycling conditions. Purified PCR products were sequenced to evaluate the presence of the AGG^Arg^→AGT^Ser^ or AGG^Arg^→AGC^Ser^ mutation at codon 249.

### iTRAQ labeling and 2DLC_ESI_MS/MS

HCC and normal hepatic tissue (0.2 g each) were ground into powder in liquid nitrogen with a precooled mortar and pestle. Samples were then homogenized on ice in 1 ml of lysis buffer (7 M urea, 2 M thiourea, 4% CHAPS, 30 mM Tris-Cl, pH 8.5, protease inhibitor mixture) using a glass homogenizer. After sonication on ice for 10 seconds using an ultrasonic processor, the samples were centrifuged for 30 min at 12,000 rpm to remove particulate materials. Protein concentrations were determined in duplicate by the Bradford method (Bio-Rad) and confirmed by SDS-PAGE.

The extracted proteins from samples within each group (i.e., HBV(+)/AB1(+) HBV(+)/AFB1(-), HBV(-)/AFB1(+), and HBV(-)/AFB1(-) were precipitated with isopropanol, and pellets were redissolved in the dissolution buffer (0.5M triethylammonium bicarbonate, 0.1% SDS). The subsequent protein samples were quantified and 100 µg of protein was denatured, alkylated, and digested.

Proteins were labeled with the iTRAQ tags as follows: normal liver tissues: 113 isobaric tag, HBV(+)/AFB1(+) group: 116 isobaric tag, HBV(+)/AFB1(-) group: 117 isobaric tag, HBV(-)/AFB1(+) group: 118 isobaric tag, HBV(-)/AFB1(-) group:119 isobaric tag. The labeled samples were combined, desalted with Sep-Pak Vac C18 cartridge 1 cm3/50mg (Waters, Milford Massachusetts USA), and fractionated by using a Shimazu UFLC system (Shimadzu Corp, Kyoto Japan) connected to a strong cation exchange (SCX) column (polysulfethyl column, 2.1mm-100 mm, 5 uL, 200 Å, (The Nest Group Inc., Southborough, MA USA). 

SCX separation was performed using a linear binary gradient of 0-45% buffer B (350mM KCl, 10mM KH_2_PO_4_ in 25% ACN, pH 2.6) in buffer A (10mM KH_2_PO_4_ in 25% ACN, pH2.6) at a flow rate of 200 uL/min for 90 min, and 30 fractions were collected every 3 min. Each fraction was dried down and redissolved in buffer C (5% (v/v) acetonitrile and 0.1% formic acid solution), and the fractions with high KCl concentration were desalted with PepClean C-18 spin columns (Pierce, Waltham USA). All SCX fractions were analyzed 3 times using a QSTAR XL LC/MS/MS system (Applied Biosystems, Foster City, California USA) and RPLC column (ZORBAX 300SB-C18 column, 5 μm, 300 Å, 0.1 mm -15 mm [Microm, Auburn, CA US]). The RPLC gradient was 5% to 35% buffer D (95% acetonitrile, 0.1% formic acid) in buffer C at a flow rate of 0.3 uL/min in 120 min. 

The Q-TOF instrument was operated in positive ion mode with ion spray voltage typically maintained at 2.0 kV. Mass spectra of iTRAQ labeled samples were acquired in an information-dependent acquisition mode. The analytical cycle consisted of a MS survey scan (400-2000 m/z) followed by 5-s MS/MS scans (50-2000) of the 5 most abundant peaks (i.e., precursor ions), which were selected from the MS survey scan. Precursor ion selection was based upon ion intensity (peptide signal intensity above 25 counts/s) and charge state (2+ to 4+). Once the ions were fragmented in the MS/MS scan, they were allowed one repetition before a dynamic exclusion for a period of 120 sec. Because of the iTRAQ tags, the parameters for rolling collision energy were manually optimized. Under CID, iTRAQ-labeled peptides fragmented to produce reporter ions at 113.1, 116.1, 117.1, 118.1 and 119.1, and fragment ions of the peptides were simultaneously produced, resulting in sequencing of the labeled peptides and identification of the corresponding proteins. The ratios of the peak areas of the three iTRAQ reporter ions reflected the relative abundances of the peptides and the proteins in the samples. Calibration of the mass spectrometer was carried out using BSA tryptic peptides.

### Database Searching and Criteria

Protein identification and quantification for the iTRAQ experiment was performed with the ProteinPilot software version 3.0 (Applied Biosystems). The Paragon Algorithm in ProteinPilot software was used for peptide identification and isoform specific quantification. 

To minimize false positive results, a strict cutoff for protein identification was applied with the unused ProtScore g1.3, which corresponds to a confidence limit of 95%, and at least one peptide with the 95% confidence was considered for protein quantification. The resulting data set was auto bias corrected to remove any variations due to unequal mixing during the combining of different labeled samples. For iTRAQ quantitation, the peptide for quantification was automatically selected by the Pro Group algorithm (at least one peptide with 99% confidence) to calculate the reporter peak area, error factor (EF), and *P*-value.

### AKR1B10 mRNA and Western blot analysis

To validate differential expression indicated by the aCGH and proteomic analysis (see above), the levels of RNA and protein of 157 samples from this study were analyzed by RT-PCR and Western blot.

Total RNA was extracted with RNAsimple Total RNA Kit (Tiangen Biotech, Beijing, China) and purified with the RNeasy Mini kit (Qiagen) according to the manufacturers’ instructions. cDNA was synthesized with the RevertAid™ First Strand cDNA Synthesis Kit (Thermo Scientific ) according to the manufacturer’s instructions. AKR1B10 cDNA was amplified using the forward and reverse primers 5'-cccaaagatgataaaggtaatgccatcggt-3’ and 5'-cgatctggaagtggctgaaattggaga-3’, respectively. GAPDH cDNA was amplified as a control using the forward and reverse primers 5’-atgaccccttcattgacc-3’ and 5’-gaagatggtgatgggatttc-3’, respectively. All cDNA amplification was peformed using standard conditions. 

For Western analysis, pooled samples used in the iTRAQ experiment that contained 20μg of total proteins were separated by 10% (w/v) SDS-PAGE and transferred onto a PVDF membrane (Millipore, USA). Blots were incubated with anti-AKR1B10 antibodies (1:1500 dilution; Abcam, Cambridge, MA, USA) and subsequently incubated with anti-mouse or anti-rabbit horseradish peroxidase-conjugated antibody. Proteins were visualized with ECL Western blotting detection reagents (Pierce Thermo Scientific) and bands quantified using QUANTITY ONE software (Bio-Rad, Hercules, CA, USA).

### Statistical analysis

Continuous variables were presented as median and inter-quartile range due to the small sample size. AKR1B10 mRNA and protein expression level in the four HBV/AFB1 exposure groups were compared by one-way ANOVA with LSD post-hoc tests. The association of 25 recurrently altered regions (RARs) with histological grade, TNM stage, and HBV/AFB1 exposure groups were assessed by Fisher’s exact test, and Benjamini and Hochberg procedure was used for control false discovery rate (FDR). Cox proportional hazard models were performed to determine independent influence factors of tumor-free survival. Variables statistically significant in univariable analyses were stepwise entered into the multivariable analysis. Statistical analyses were two-sided and *P*-value < 0.05 was considered statistically significant. Statistical analyses were performed using SPSS 15.0 software (SPSS, Chicago, IL).

## Results

### Baseline demographics and disease characteristics of 32 patients for aCGH and iTRAQ analysis

All 32 patients were categorized into 4 groups depending upon their HBV and AFB1 status. The categories were: HBV(+)/AFB1(+) group (n = 10); HBV(+)/AFB1(-) group (n = 10); HBV(-)/AFB1(+) (n = 6); and HBV(-)/AFB1(-) (n = 6). The average age of the study population was about 50 years, most were male (93.8%), most had liver cirrhosis (87.5%), and about half were HBV and AFB1 positive. About 53.1% of patients had Edmondson grade of II and 56.3% of patients had TNM stage of I+II. There was no significant difference in clinical characteristics between groups. (Table S1). A total of 23 patients had a long history of smoking. Of these patients, 7 were in the HBV (+) / AFB1 (+) group, 8 were in the HBV (+) / AFB1 (-) group, 5 were in the HBV (-) / AFB1 (+) group and 6 were in the HBV (+) / AFB1 (+) group. None of the patients had a family history of liver cancer or a history of drinking (all patients were negative for long-term exposure to alcohol).Tumor-free rate was defined as the percentage of study subjects without recurrence of the tumor at given time points after surgery. The six-month, 1-year, and 2-year tumor-free rates were 56.2%, 35.8%, and 21.8%. 

### Chromosomal alterations in patients with HCC

**Figure 1:**
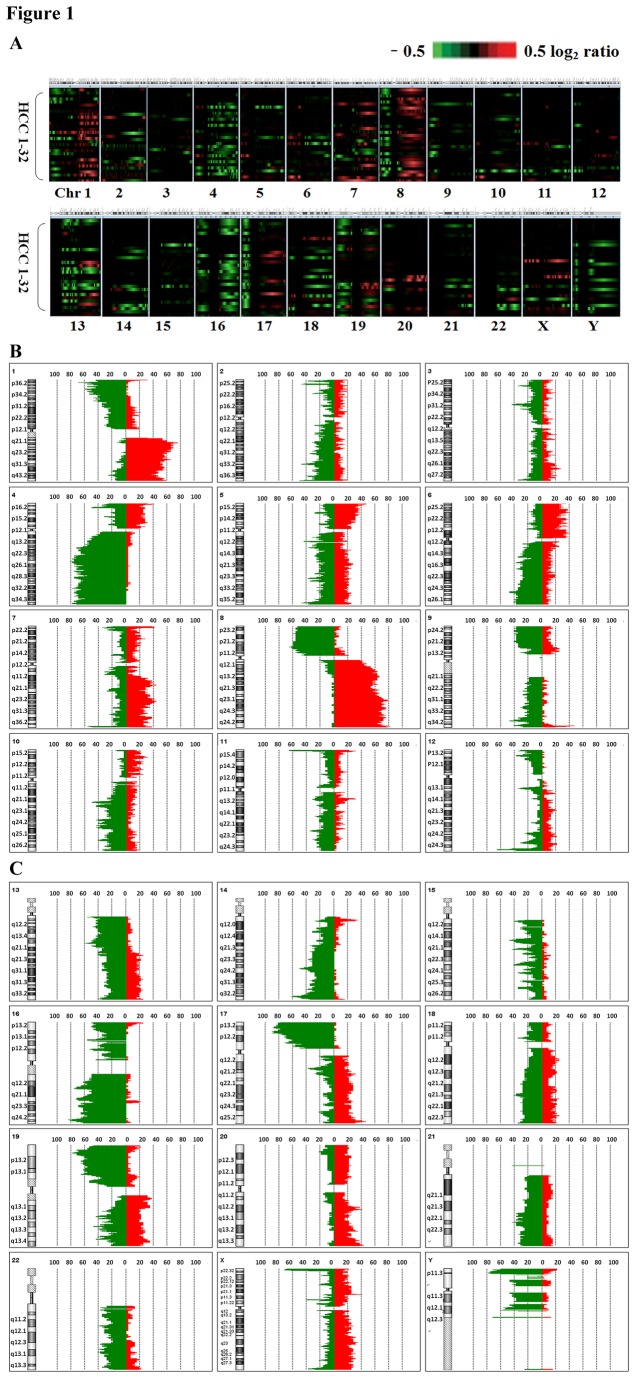
Whole-genome profiles and frequency plots of chromosomal alterations in HCC patients (z-scoring:2.5). (A) The genomic alterations in tumor samples from each HCC patient (n = 32) are illustrated in individual vertical lanes. A total of 573 gene copy number alterations were mapped and ordered by chromosomal position from 1pter to Yqter using the workbench Lite Edition 6.5.0.18. Tumor versus the reference intensity ratios (in log_2_ ratio) for individual tumor samples are plotted in different color scales reflecting the extent of increases (red) or decreases (green) in copy number. The detail chromosomal alterations were shown in 1B (chromosome 1 to chromosome 12) and in 1C (chromosome 13 to chromosome 22, X chromosome and Y chromosome, respectively). Nine chromosomes showed increased chromosomal DNA (1q, 5p, 6p,7q, 8q, 17q, 20p, 20q and X) and 16 chromosomes had deceased chromosomal DNA (1p, 4q, 8p, 9p, 10q,13q, 14q, 16p, 16q ,17p ,18q, 19p ,19q, 21q, 22q and Y) that were repeatedly observed in > 20% of tumor samples.

Analysis of chromosomal alterations indicated that chromosomal abnormalities were observed in the tumors of the HCC patients (Figure 1). Chromosomal instability was not equally distributed across all the chromosomes. Of a total of 573 chromosomal aberrations, 184 resulted in increased (gains) and 389 in decreased (losses) genetic material. The mean gains or losses of genetic material per patient were 5.7 and 12.2, respectively. Genetic alterations across chromosomal arms were detected in at least 7 tumors (21.9%) including increased chromosomal DNA in 1q, 4p, 5p, 6p,7p, 8q, 10p, 17q, 20p, 20q and X and decreased chromosomal DNA in 1p, 2q, 4q, 8p, 9p, 10q, 11q, 13q, 14q, 16p, 16q ,17p ,19p ,19q, 21q, 22q ,Y (Figure 1).

### Recurrently altered regions (RARs) in HCC tumors

To identify genetic regions that were sensitive to genetic alterations, we defined the recurrently altered regions (RARs) as regional chromosomal alterations observed in tumors from ≥10 patients. We detected a total of 25 RARs (including 8 RARs that increased and 17 RARs that decreased in copy number) (Table 1). These regions contain a number of oncogenes (e.g., *MYC, FGF, EGFR* and *CCND3*), tumor suppressor genes (e.g., *TP53, PTEN, RB1, BRCA2, CDKN2A* and *CDKN2B*), detoxification and drug metabolism genes (e.g.*, GSTA1, ADH4, ADH5, ADH6, ADH7, ADH1A, ADH1B, ADH1C, CYP27A1, EPHX1, EPHX2*, *AKR1B10, AKR7A2 and AOX1*) as well as genes involved in a number of other cellular processes (Table 1). 

**Table 1:** Recurrently altered regions (RARs) arranged by chromosomal location (Z-score: 2.5|log2ratio|>0.225).

Chromosome Position	Change in chromosomal DNA (Gain/Loss)	Number of cases	Previously Reported Cancer-related Genes
1p31.2-p36.2	loss	16	*AKR7A2, PRDM2, RIZ, RAD54L, FAF1, STIL, CDKN2C, TTC4, JUN, ARHI, PRDM2, RIZ, CASP9, PGM1, ENO1*
1q21.1-q44	gain	20	*PDZK1, MCL1, ARNT, AF1Q, TPM3, ADAR, RPS27, HAX1, PYGO2, CKS1B, ADAM15, MUC1, HDGF, CCT3, PRCC, IFI16, AIM2, USF1, SELP, SELE, LAMC2, TPR, PTGS2, KIF14, ELF3, MDM4, ATF3, TGFB2, WNT3A, AKT3, EPHX1*
2q23.2-q37.2	loss	11	*AOX1*, *CYP27A1*, *HSPD1*, *HSPE1*
4q13.3-q35.2	loss	22	*ADH4, ADH5, ADH6, ADH7, ADH1A, ADH1B, ADH1C, HADH, ACSL1, FGA, FGB, ACSL1 , CASP3, FAT, FSTL5, VEGFC,*
5p13.2-p15.3	gain	11	*AMACR*
6p12.1-p25.2	gain	13	*HIST1H2AA , HIST1H2AI, HIST1H2AJ, HIST1H2AK, HIST1H1B, HIST1H4A , HIST1H5A, HIST1H2BJ , HIST1H2BJ , HSP90AB1, HSPA1A , HMGA1, NOTCH4, MAPK14, PIM1, TFEB, CCND3,VEGF, GSTA1, DEK, ID4, E2F3, PRL,MICA, MICB,*
6q14.1-q26	loss	10	*CCNC, GRIK2, CRSP3, PLAGL1,SASH1, LATS1, IGF2R, UNC93A, MLLT4, GOT2*
7q11.2-q35	gain	13	*AKR1B10, HGF, DMTF1, ABCB1, EPO, EPHB4, PIK3CG, PDIA4, CAV1,CAV2, MET, WNT2, MDH2,CALD1*
8p12-p23.2	loss	19	*EPHX2, CSMD1, DEFB1, NAT1, NAT2, PSD3, TNFRSF10A, TNFRSF10B, TNFRSF10C, RHOBTB2*,
8q11.2-q24.3	gain	22	*PRKDC, MCM4, SNAI2, LYN, MOS, PLAG1, COPS5, TPD52, E2F5, MMP16, NBS1,EIF3S3, C-MYC, KCNK9, PTK2, EIF2C2, CCNE2*
9p21.1-p24.2	loss	12	*SMARCA2, MTAP, CDKN2B, CDKN2A, RECK, PAX5*
10q21.3-q26.2	loss	12	*PTEN, CYP2E1, ECHS1*
13q12.1-q21.2	loss	13	*RB1, BRCA2, XPO4,CCNA1, RFP2, DDX26, DLEU1* *DLEU2*
14q21.3-q32.2	loss	13	*AKT1, PCK2*,*PYGL* ,*HSP90AA1*
16p12.1-p13.2	loss	14	*SULT1A1, SOCS1, ERCC4, GNMT* , *ABAT*
16q12.1-q24.1	loss	21	*ARG1, CDH1, CDH3,CDH13,BCAR1, WWOX, WFDC1*
17p12-p13.3	loss	25	*TP53* , *MYH10*
17q12-q25.2	gain	10	*HLF, MPO, PPM1D, BCAS3, TBX2*
18q12.3-q22.3	gain	10	--
19p13.1-p13.3	loss	19	*CD97,DDX39, PRKCL1, EIF3S4, DNMT1, P2RY11* *KIAA1198,*
19q13.2-q13.4	loss	10	*CCNE1, APOE*
21q21.3-q22.2	loss	11	*ADAMTS1, SOD1* , *FTCD*
22q11.2-q13.2	loss	10	*COMT*
X	gain	11	*--*
Y	loss	14	--

Two chromosomal regions showed an association of instability with tumor stage. Loss of 8p12-p23.2 was significantly higher in high stage tumors compared to low stage tumors (TNM I-II: 38.9% vs. TNM III: 92.9%; *P* = 0.038) and loss of 19p13.1-p13.3 was also significantly higher in high stage tumors compared to low stage tumors (TNM I-II: 33.3% vs. TNM III: 92.9%; *P* = 0.025) (Table S2). There was no association of any RAR with Edmondson grade (all *P*-values > 0.05). 

### Association between RARs and HBV status and AFB1 exposure

**Figure 2:**
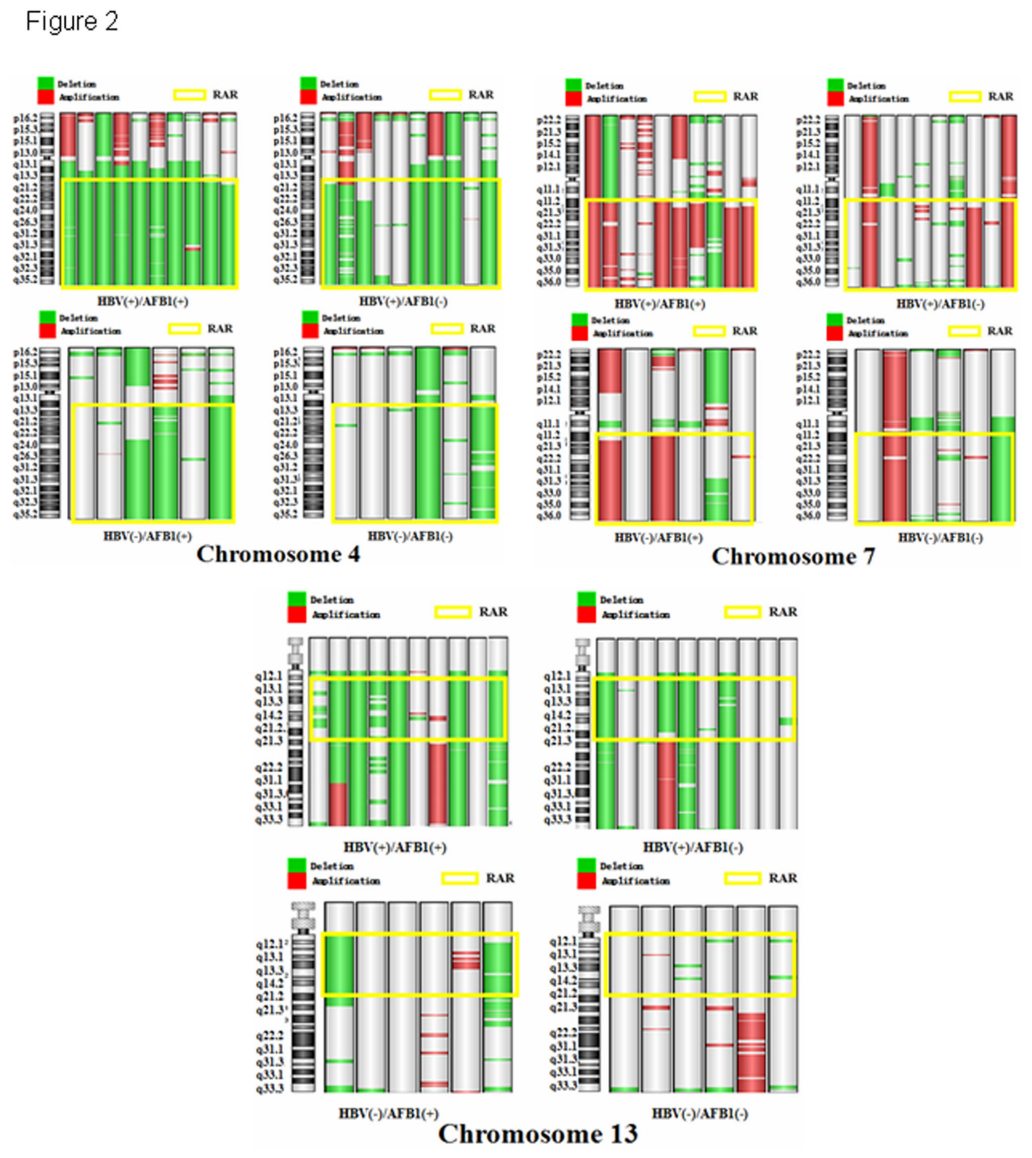
Representative comparison of incidence of RAR:4q12-q35.2、RAR:13q12.1-q21.1 and RAR:7q11.2-q35 in the 4 groups based on HBV and AFB1 status. (%). Red bar: the loss of chromosome; Green bar: the gain of chromosome. Yellow frame: Recurrently Altered Regions (RARs).

We analyzed the association of the different groups (HBV(+)/AFB1(+), HBV(+)/AFB1(-), HBV(-)/AFB1(+) and HBV(-)/AFB1(-)) with occurrence of RARs and found that all 4 groups showed a high incidence of chromosomal alterations (≥50%) at RAR: 8q11.2-q24.3, 17p12-p13.3, 19p13.1-p13.3 (Table 2). Loss of 4q13.3-q35.2, 13q12.1-q21.2, and gain of 7q11.2-q35 were observed with a higher frequency in the HBV(+)/AFB1(+), HBV(+)/AFB1(-) and HBV(-)/AFB1(+) groups compared to the HBV(-)/AFB(-) group (Figure 2). Despite the small numbers in the 4 groups, we found no statistical difference among the 4 groups in the incidence of a particular RAR. When analyzing a single factor by itself, a higher incidence of RARs was observed in 4q13.3-q35.2 among HBV-positive patients compared to HBV-negative patients (85.0% vs. 41.7%, *P* = 0.018). However, after using the Benjamini Hochberg analysis to control for false positives, this was non-significant (Table S3).

**Table 2:** The comparison of incidence of RARs in 32 HCCs by HBV and AFB1 status.

**Chromosome**	**Group**				**Adjusted P-value^*#*^**
	**HBV(+)/AFB1(+)**	**HBV(+)/AFB1(-)**	**HBV(-)/AFB1(+)**	**HBV(-)/AFB1(-)**		
	**(n=10)**	**(n=10)**	**(n=6)**	**(n=6)**		
1p31.2-p36.2	6 (60.0%)	5 (50.0%)	2 (33.3%)	3 (50.0%)	0.977
1q21.1-q44	6 (60.0%)	7 (70.0%)	2 (33.3%)	5 (83.3%)	1.000
2q23.2-q37.2	4 (40.0%)	2 (20.0%)	2 (33.3%)	3 (50.0%)	0.958
4q13.3-q35.2	10 (100.0%)	7 (70.0%)	3 (50.0%)	2 (33.3%)	0.400
5p13.2-p15.3	2 (20.0%)	4 (40.0%)	1 (16.7%)	4 (66.7%)	1.000
6p12.1-p25.2	3 (30.0%)	3 (30.0%)	4 (66.7%)	3 (50.0%)	1.000
6q14.1-q26	2 (20.0%)	4 (40.0%)	1 (16.7%)	3 (50.0%)	1.000
7q11.2-q35	7 (70.0%)	3 (30.0%)	2 (33.3%)	1 (16.7%)	1.000
8p12-p23.2	8 (80.0%)	4 (40.0%)	3 (50.0%)	5 (83.3%)	1.000
**8q11.2-q24.3^*▲*^**	**7 (70.0%)**	**6 (60.0%)**	**6 (100.0%)**	**3 (50.0%)**	1.000
9p21.1-p24.2	5 (50.0%)	3 (30.0%)	2 (33.3%)	2 (33.3%)	0.934
10q21.3-q26.2	5 (50.0%)	3 (30.0%)	3 (50.0%)	1 (16.7%)	0.953
13q12.1-q21.2	7 (70.0%)	4 (40.0%)	2 (33.3%)	0 (0.0%)	0.613
14q21.3-q32.2	5 (50.0%)	3 (30.0%)	2 (33.3%)	3 (50.0%)	0.996
16p12.1-p13.2	4 (40.0%)	5 (50.0%)	2 (33.3%)	3 (50.0%)	0.963
16q12.1-q24.1	7 (70.0%)	7 (70.0%)	2 (33.3%)	5 (83.3%)	1.000
**17p12-p13.3^*▲*^**	**10 (100.0%)**	**7 (70.0%)**	**4 (66.7%)**	**4 (66.7%)**	1.000
17q12-q25,2	5 (50.0%)	3 (30.0%)	1 (16.7%)	1 (16.7%)	1.000
18q12.3-q22.3	4 (40.0%)	2 (20.0%)	3 (50.0%)	1 (16.7%)	1.000
**19p13.1-p13.3^*▲*^**	**7 (70.0%)**	**5 (50.0%)**	**4 (66.7%)**	**3 (50.0%)**	0.996
19q13.2-q13.4	4 (40.0%)	4 (40.0%)	1 (16.7%)	1 (16.7%)	0.920
21q21.3-q22.2	2 (20.0%)	4 (40.0%)	3 (50.0%)	2 (33.3%)	0.958
22q11.2-q13.2	4 (40.0%)	1 (10.0%)	2 (33.3%)	3 (50.0%)	1.000
X	3 (30.0%)	2 (20.0%)	3 (50.0%)	3 (50.0%)	0.950
Y^※^	4 (44.4%)	3 (33.3%)	4 (66.7%)	3 (50.0%)	0.926

^#^ Benjamini and Hochberg procedure was used for control false discovery rate (FDR).

^▲^ High incidence of chromosomal alterations (≥50%) in all four groups are highlighted in bold.

***^※^***1 out of 10 patients is female in HBV(+)/AFB1(+) and HBV(+)/AFB1(-) groups, respectively.

### Univariate and multivariate analysis of patient characteristics and RARs with tumor-free survival

Univariate analysis found an association of tumor-free survival with tumor size, serum AFP ≥ 400 ng/mL, BCLC, TNM stage, invasion and metastasis, and loss of 8p12-p23.2 and 19p13.1-p13.3 (all *P*-values ≤ 0.025) (Table 3). Multivariate analysis showed that serum AFP (≥400), TNM stage, loss of 8p12-p23.2 were independent factors associated with tumor-free survival (all *P*-values ≤ 0.045) (Table 3).

**Table 3:** Univariate and multivariate analysis of patient characteristics and RARs with tumor-free survival using COX regression models.

**Variables**	**Univariable**			**Multivariable**	
	**OR (95% CI)**	**P-value**		**OR (95% CI)**	**P-value**
**Age (year)**	1.00 (0.96, 1.04)	0.869			
**Gender**	1.84 (0.25, 13.75)	0.552			
**Tumor size (>3cm**)	1.30 (1.12, 1.50)	<0.001*			
**AFP > 400 ng/ml**	4.08 (1.35, 12.32)	0.013*		7.87 (2.25, 27.57)	0.001*
**HBV**	1.00 (0.43, 2.31)	0.994			
**AFB1**	1.80 (0.78, 4.15)	0.169			
**Liver cirrhosis**	32.24 (0.45, 2,307.29)	0.111			
**BCLC grade**					
**A**	Reference				
**B**	8.30 (2.17, 31.82)	0.002*			
**C**	7.90 (2.49, 25.04)	<0.001*			
**Edmondson grade**	2.22 (0.94, 5.25)	0.071			
**TNM stage**	6.65 (2.50, 17.73)	<0.001*		6.70 (2.00, 22.42)	0.002*
**Invasion and metastasis**	7.62 (2.64, 21.99)	<0.001*			
**1p31.2-p36.2**	0.73 (0.32, 1.64)	0.444			
**1q21.1-q44**	0.94 (0.41, 2.15)	0.875			
**2q23.2-q37.2**	0.86 (0.35, 2.08)	0.736			
**4q13.3-q35.2**	0.60 (0.25, 1.42)	0.242			
**5p13.2-p15.3**	0.92 (0.38, 2.22)	0.857			
**6p12.1-p25.2**	0.99 (0.43, 2.29)	0.976			
**6q14.1-q26**	0.89 (0.36, 2.17)	0.791			
**7q11.2-q35**	0.83 (0.36, 1.92)	0.662			
**8p12-p23.2**	3.71 (1.40, 9.81)	0.008*		3.29 (1.03, 10.52)	0.045*
**8q11.2-q24.3**	1.03 (0.44, 2.41)	0.954			
**9p21.1-p24.2**	0.58 (0.24, 1.41)	0.232			
**10q21.3-q26.2**	1.02 (0.45, 2.34)	0.962			
**13q12.1-q21.2**	1.75 (0.77, 4.00)	0.184			
**14q21.3-q32.2**	0.81 (0.34, 1.94)	0.631			
**16p12.1-p13.2**	1.04 (0.45, 2.38)	0.928			
**16q12.1-q24.1**	0.83 (0.36, 1.89)	0.652			
**17p12-p13.3**	1.23 (0.45, 3.33)	0.690			
**17q12-q25,2**	1.54 (0.64, 3.67)	0.333			
**18q12.3-q22.3**	2.30 (0.94, 5.61)	0.069			
**19p13.1-p13.3**	2.86 (1.14, 7.15)	0.025*			
**19q13.2-q13.4**	0.85 (0.35, 2.07)	0.718			
**21q21.3-q22.2**	1.35 (0.59, 3.09)	0.483			
**22q11.2-q13.2**	1.19 (0.51, 2.81)	0.689			
**X**	0.98 (0.41, 2.34)	0.969			
**Y**	1.02 (0.45, 2.30)	0.971			

^*^ Indicates a significant association with tumor-free survival

### iTRAQ Analysis of differentially expressed proteins in HCC tumors

Using mass spectrophotometry, we identified differentially expressed proteins by the following criteria: unused protein score was more than 1.3 (99% confidence) per experiment; one or more peptide hits were found per protein at > 95% confidence per peptide, and the ratio of expression in HCC tumors versus control liver tissue differed by at least 2-fold in the total population or 0.5-fold in the four sub-groups. After meeting these criteria the identified proteins (n = 649) were subsequently filtered with manually selected filter exclusion parameters. 

**Figure 3:**
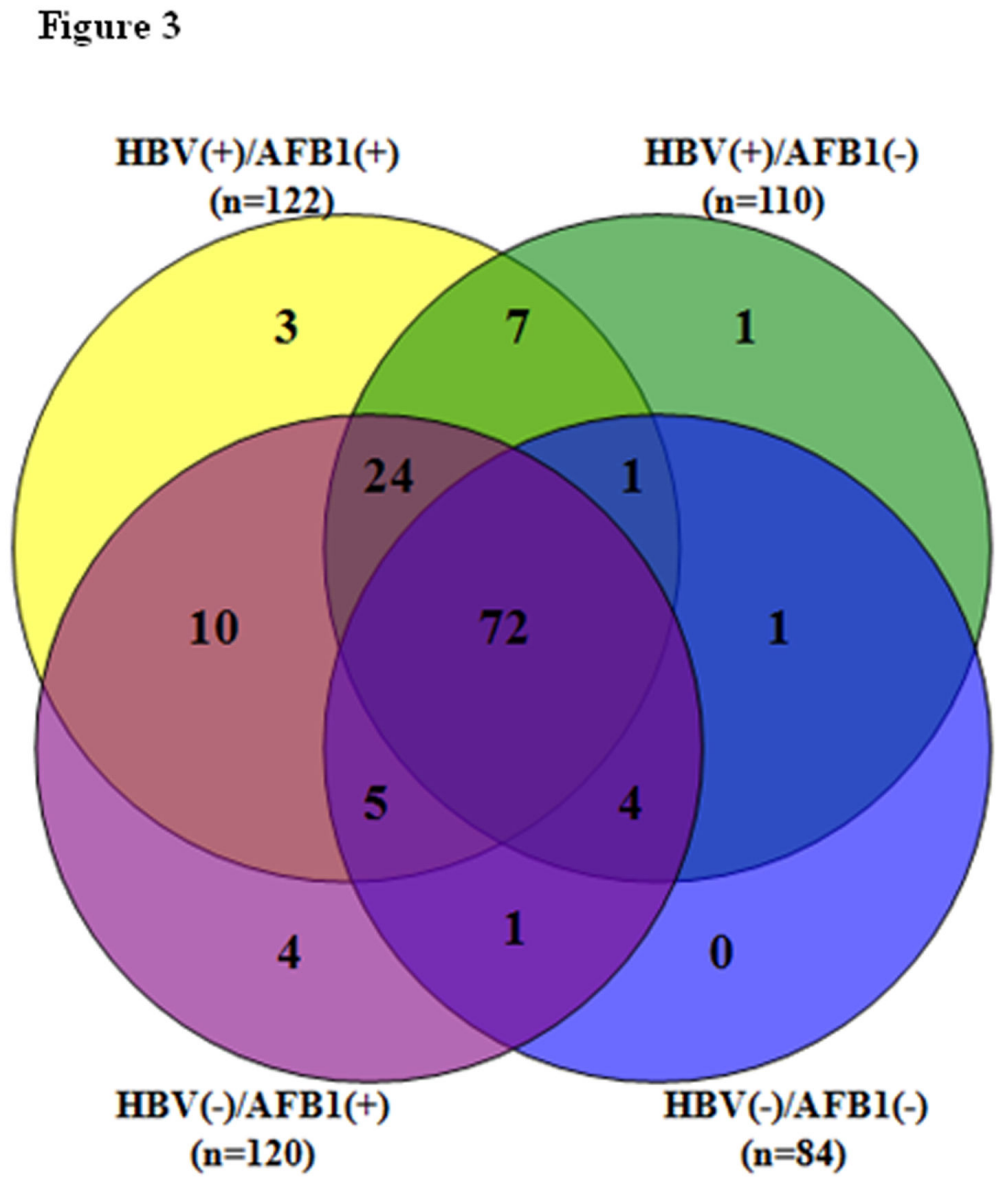
Venn diagram depicting the overlap of differentially expressed proteins quantified in the 4 groups based on HBV and AFB1 status. The number in parentheses indicates the number of differentially expressed quantified proteins in each group.

We identified a total of 133 differentially expressed tumor proteins (59 proteins were significantly up-regulated and 74 proteins were significantly down-regulated) relative to control liver tissue in the four groups (122, 110, 120 and 84 proteins in HBV(+)/AFB1(+), HBV(+)/AFB1(-), HBV(-)/AFB1(+) and HBV(-)/AFB1(-) groups, respectively) (Figure 3 and Table S4). Of these 133 proteins, 72 were commonly up-regulated or down-regulated in all of the 4 subgroups, and 24 were commonly changed in HBV(+)/AFB1(+), HBV(+)/AFB1(-), and HBV(-)/AFB1(+) groups (Figure 3). We found that 69 (51.8%) of the 133 differentially expressed proteins mapped within the 25 identified RARs, and a number of them (*AKR1B10, AKR7A2, ENO1, EPHX2, ADH1, ADH1C, ADH1A, ADH6, GSTA1, APOE, HSP90AA1, GNMT, COMT, HIST1H2AA, HIST1H1B* , *ARG1*, ect ) are cancer-related genes (Table 1 and Table S3). 

Using DAVID (http://david.abcc.ncifcrf.gov) to classify the molecular processes that may be affected by the altered protein expression, we showed that 62 of the 133 differentially expressed proteins were involved in 13 biological pathways. These included detoxification and drug metabolism; antigen processing and presentation; glycolysis; anti-apoptosis; response to unfolded protein; macromolecular complex assembly; steroid metabolic process; coenzyme metabolic process; homeostasis in body; fatty acid metabolic process; response to nutrient levels; circulatory system process and cytoskeleton organization (Table 4). 

**Table 4:** Biological process categories of differentially expressed proteins.

**Category**	***P*-Value^*#*^**	**Term (No.)**	**Gene Name^*▲*^**
**Detoxification and drug metabolism pathway**	1.22E-6	GO:000098(2)	*GSTA1, CYP2A13, ADH4, ADH1C, CYP2E1, ADH1A, ADH6, EPHX2, EPHX1, AKR1C1, AKR1C4, AKR1B10, AKR7A2，AOX1, HADH, CYP27A1.*
**Antigen processing and presentation pathway**	0.002541	GO:000645(7)	*HSP90AB1, HSP90AA1, PDIA3, HSPA1A, HSPA5, CALR, CANX, HSPA8, GPP78, GRP75, HSPD1, VCP,*
**Glycolysis pathway**	6.32E-7	GO:000609(6)	*LDHA, PKM2, ALDOB, PGM1, PGK1, MDH2, ENO1*
**Anti-apoptosis pathway**	5.68E-4	GO:000691(6)	*HSP90AB1, HSP90AA1, APOE, CFL1, TGM2, HSPB1, HSPA1A, GRP75, HSPD1, SOD1, RPS27A, GRP78, HSPE1*
**Fatty acid metabolic process pathway**	0.010114	GO:000663(1)	*ACAA2, CRYL1, ACSL1, EPHX2, HADH, SLC27A5*
**Response to unfolded protein**	4.29E-7	GO:000698(6)	*HSP90AB1, HSP90AA1, VCP, HSPB1, HSPE1, HSPA1A, HSPD1, HSPA8*
**Macromolecular complex assembly**	1.51E-6	GO:006500(3)	*HSP90AA1, OTC, ALDOB, SLC9A3R1, CALR, FLNA, HIST1H4A, FGA, VCP, FGB, APOE, HIST1H2BJ, TGM2, TOMM22, GNMT, HSPD1, TUBA1B, AKR1C1, HIST1H2AA,*
**Steroid metabolic process**	3.91E-4	GO:000820(2)	*ACAA2, AKR1C4, CYP27A1, APOE, AKR1B10, COMT, AKR1C1, SLC27A5*
**Coenzyme metabolic process**	7.03E-5	GO:000673(2)	*MTHFD1, GSTA1, ALDH1L1, ALDOB, FTCD, SOD1, DCXR, MDH2*
**Homeostasis in body**	0.020564	GO:004887(8)	*PYGL, APOE, OTC, TXN, EPHX2, TGM2, PDIA6, PDIA4, SOD1, SLC9A3R1, CALR, AKR1C1*
**Response to nutrient levels**	0.010081	GO:003166(7)	*ACSL1, ALDOB, CTSD, HSPA5, COL1A1, SOD1*
**Circulatory system process**	0.035458	GO:000301(3)	*APOE, EPHX2, ABAT, SOD1, HBB*
**Cytoskeleton organization**	0.026019	GO:000701(0	*APOE, CALD1, CFL1, SOD1, CALR, TUBA1B, FLNA, MYH10*

^#^ Significance level of enrichment was calculated using hypergeometric distribution and P<0.05 was considered significant.

^▲^ Gene symbols for the differentially expressed proteins

### AKR1B10 mRNA and protein analysis

**Figure 4:**
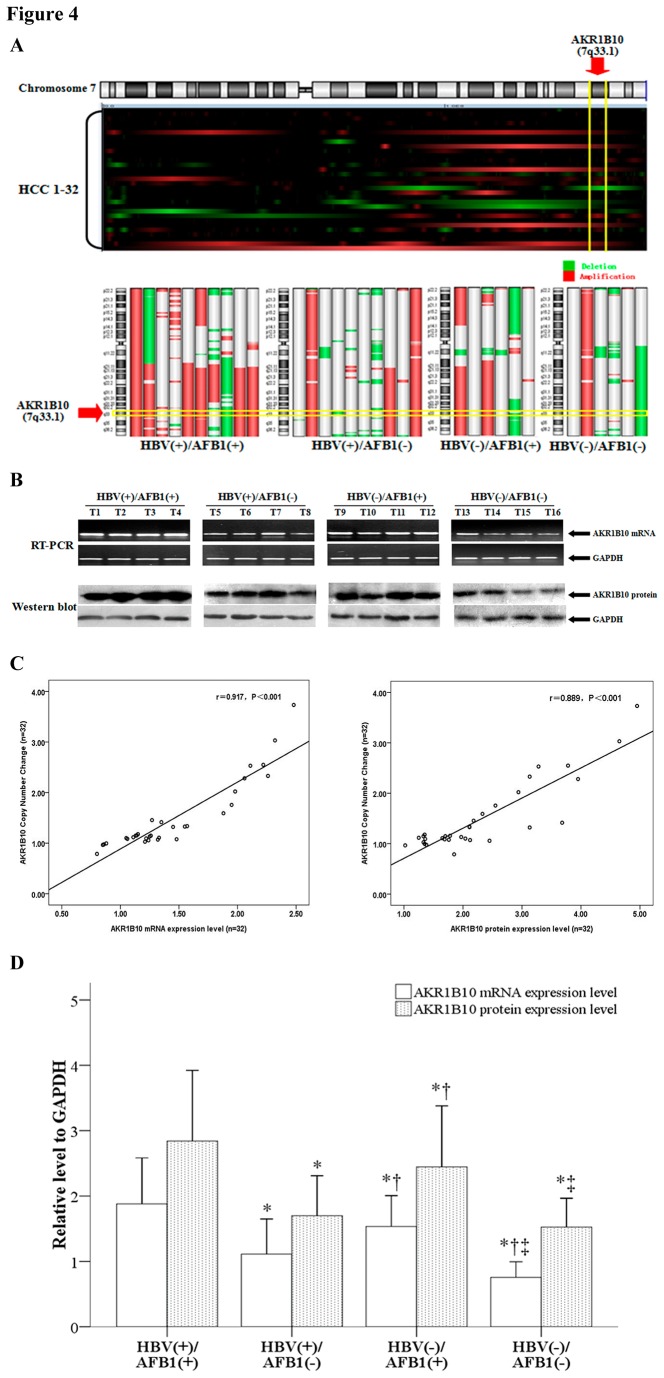
The AKR1B10 gene location and its expression levels validated by Reverse Transcriptase PCR and Western Blot. (A) AKR1B10 is located on RAR: 7q11.2-q35 (position 7q33.1) a chromosomal region that showed a high instance of instability in HBV(+)/AFB1(+) ,HBV(+)/AFB1(-) and HBV(-)/AFB1(+) groups. (B) Representative RT- PCR and Western blot images of AKR1B10 in the 4 groups. T1–T4 ,T5-T8, T9-T12 and T13-T16 were HCC samples obtained from HBV(+)/AFB1(+) , HBV(+)/AFB1(-), HBV(-)/AFB1(+) and HBV(-)/AFB1(-) groups, respectively. Human GAPDH was used as an internal control. (C) The regression analysis showed a significant correlation between AKR1B10 expression level (both mRNA level and protein level) and AKR1B10 copy number. (D) RT-PCR and Western blot analysis of AKR1B10 expression. Mean mRNA and protein expression levels of AKR1B10 were calculated by RT-PCR and Western blotting for the 4 subgroups. Human GAPDH was used as an internal control. * indicated a significant difference (*P*<0.05) as compared to HBV(+)/AFB1(+), †indicated a significant difference (*P*<0.05) as compared to HBV(+)/AFB1(-), ‡ indicated a significant difference (*P*<0.05) as compared to HBV(-)/AFB1(+).

One protein that was differentially expressed among the 4 patient groups was aldo-keto reductase family 1 member B10 (AKR1B10); AKR1B10 protein levels were high in HBV(+)/AFB1(+) and HBV(-)/AFB1(+) groups (9.22 and 5.15, respectively) and lower in HBV(+)/AFB1(-) and HBV(-)/AFB1(-) groups (2.00 and 1.10, respectively). AKR1B10 is located on RAR: 7q11.2-q35 (position 7q33.1), a chromosomal region that showed a high instance of instability (Figure 4A). 

The AKR1B10 mRNA and protein expression levels in 32 HCC tumor samples and correlations among the expression of AKR1B10 mRNA, protein levels and AKR1B10 copy number were investigated first. The regression analysis showed a significant correlation between the expression of AKR1B10 mRNA and protein levels and AKR1B10 copy number (Figure 4B, 4C). A total of 157 samples comprising (HBV(+)/AFB1(+) (n=52), HBV(+)/AFB1(-) (n=50), HBV(-)/AFB1(+) (n=30) and HBV(-)/AFB1(-) (n=25)) were subjected to RT-PCR and Western blot analysis in order to further analyze the association between AKR1B10 expression and HBV status and AFB1 exposure. 

The mean expression level of AKR1B10 mRNA was significantly higher in the HBV(+)/AFB1(+) group compared to the other three groups (1.88 vs. 1.11, 1.53, and 0.76, *P* ≤ 0.008). Samples from the HBV(-)/AFB1(+) group also showed significantly higher AKR1B10 mRNA levels compared to the HBV(-)/AFB1(-) group (1.53 vs. 0.76, *P*<0.001) and the HBV(+)/AFB1(-) group (1.53 vs. 1.11, *P* = 0.001). In addition, the HBV(+)/AFB1(-) group also showed significantly higher AKR1B10 mRNA levels than the HBV(-)/AFB1(-) group (1.11 vs. 0.76, *P* = 0.01). AKR1B10 protein was expressed at significantly higher levels in the HBV(+)/AFB1(+) group compared to the other three groups (2.84 vs. 1.70, 2.45, and 1.53 *P* ≤ 0.041). Samples from the HBV(-)/AFB1(+) group showed significantly higher AKR1B10 protein levels than the HBV(-)/AFB1(-) group (2.45 vs. 1.53, *P* < 0.001) and the HBV(+)/AFB1(-) group (2.45 vs. 1.70, *P* < 0.001) (Figure 4B ,4D)., 

## Discussion

A variety of risk factors have been associated with HCC. HBV infection and AFB1 exposure contribute to HCC development in more than 80% of the HCC cases in the Guangxi area of Southern China [4]. Given that development of HCC is a complex process associated with the accumulation of genetic changes, we examined whether patients exposed to HBV or AFB1 had specific molecular changes that may give insight to HCC tumorigenesis.

We found that chromosomal instability was not equally distributed across all the chromosomes in all patients. We found a total of 573 chromosomal abnormalities in our study patients. Of these, 184 resulted in increased and 389 in decreased genetic material with a mean gain or loss of 5.7 and 12.2 per patient, respectively. Twenty-five RARs were identified with a high incidence (≥50% of tumors) of chromosomal alterations at RARs: 8q11.2-q24.3, 17p12-p13.3, 19p13.1-p13.3. Loss of 8p12-p23.2 and loss of 19p13.1-p13.3 were associated with high TNM III stage tumors (92.9% and 92.9%, respectively). A total of 133 differentially expressed proteins were identified, 72 of which were commonly up-regulated or down-regulated in all of the 4 subgroups, and 24 of which were commonly changed in HBV(+)/AFB1(+), HBV(+)/AFB1(-), and HBV(-)/AFB1(+) groups. Of the 133 differentially expressed proteins, 51.8% mapped within identified RARs. The most common biological processes affected by HBV and AFB1 status were detoxification and drug metabolism pathways, antigen processing, glycolysis, and anti-apoptosis pathways. Analysis of AKR1B10 expression indicated that changes in its mRNA and protein expression were influenced by AFB1 exposure. These data identify a number genetic and gene expression alterations associated with HBV- and AFB1- related HCC and may give insight into HCC tumorigenesis. A strength of this study was the availability of a unique population of HCC patients who were exposed to both hepatitis viruses and AFB1 [7]. 

Previous studies have investigated the genetic abnormalities of HCC including screening for chromosomal regions with frequent allelic imbalance, comparative genomic hybridization, analysis of differential gene and protein expression, and changes in microRNA profiles [15–20,23,24]. Pathways that have been implicated in HCC tumorigenesis include P53/ARF, RB/INK4A, and WNT/ß-catenin pathways. Other genetic changes include chromosomal alterations and abnormal methylation of the promoters of tumor suppressor genes and repetitive sequences [16–18,20,23,24]. The methylation of repetitive sequences may influence chromosomal stability, and activate retrotransposons and microRNA expression. 

Chromosomal alterations are a major factor leading to the development of HCC. By utilizing array-based comparative genomic hybridization (aCGH) technology with the capacity to screen for DNA copy changes across the entire genome at high resolution, we identified a large number of chromosomal abnormalities in HCC cases from the Guangxi area. Of the chromosomal alterations detected, we showed that 21 of the 25, including the 10 most frequent RARs (1p31.2-p36.2, 1q21.1-q44, 4q13.3-q35.2, 7q11.2-q35, 8p12-p23.2, 8q11.2-q24.3, 13q12.1-q21.2, 16p12.1-p13.2, 16q12.1-q24.1, 17p12-p13.3) were consistent with those reported in previous studies [16–18,20]. Not all of the previous studies investigated the association of these abnormalities with HBV or AFB1 exposure, suggesting that the commonality between ours and these previous studies reflect aspects of HCC tumorigenesis that are independent of HBV or AFB1 exposure. 

Three RARs (2q23.2-q37.2; 18q12.3-q22.3; 19p13.1-p13.3) identified in this study have rarely been reported in previous studies. It is important to note that loss of 19p13.1-p13.3 was present in > 50% of the cases, suggesting it is unlikely to be a false positive, and that it may be a genetic characteristic of HCC from Guangxi area. These 3 RARs were similar across the 4 patient subgroups, suggesting that HBV and AFB1 likely do not have a strong affect in inducing these chromosomal abnormalities, and that there may be other environmental or genetic factor(s) present within the Guangxi area that is driving these chromosomal instabilities. At this time, it is not clear if these instabilities directly influence HCC development or are a consequence of the disease. The idea that 8p12-p23.2 could be a consequence of the disease arises from the finding that the frequency of 8p12-p23.2 loss was significantly associated with late stage tumors and was an independent factor associated with tumor-free survival. This idea is also supported by a previous study that found deletion of chromosome 8p was associated with HCC metastasis [25]. 

Although not statistically significant, we found that loss of 4q13.3-q35.2, 13q12.1-q21.2, as well as gain of 7q11.2-q35 was observed with a higher frequency in the HBV(+)/AFB1(+), HBV(+)/AFB1(-) and HBV(-)/AFB1(+) groups compared to the HBV(-)/AFB(-) group, suggesting that genes covered by these 3 RARs may play a role in HBV- and/or AFB1-related HCC carcinogenesis. The lack of statistical significance could be due to the small sample size or due to the fact that HBV or AFB1 trigger chromosome instability randomly, and do not induce specific chromosomal gain or loss. However, our findings were consistent with a study that investigated HCC patients from the Shanghai area where there was high exposure to HBV and AFB1 that found a higher frequency of loss of 4q and 13q in tumors in HCC patients compared with Hong Kong, Japan, and the United States [26]. Several other studies have demonstrated that HBV-related chromosomal rearrangements map to 4q [27–29]. Loss of 4q and 13q was also reported to correlate with the etiology of HBV-related HCC tumorigenesis [30]. Based on our data and these studies, we suggest that despite our small sample size, our findings indicated that the genetic alterations were in fact associated with HBV and AFB1- related HCC. To our knowledge, our study is the first report of gain in 7q (7q11.2-q35) in AFB1-related HCC patients. We recognize that it will be important to validate our data using larger sample sizes. 

We identified 133 proteins whose expression levels were significantly different between normal liver and HCC tissues. Sixty nine (51.8%) of these proteins mapped within RARs which implied that the genomic DNA copy number alterations, amplification and loss, could partially contribute to the dysregulation of these proteins. Of the 133 differentially expressed proteins, 16 proteins (*GSTA1, CYP2A13, ADH4, ADH1C, HADH, CYP2E1, CYP27A1，ADH1A, ADH6, EPHX2, EPHX1, AKR7A2, AKR1C1, AKR1C4, AKR1B10, and AOX1*) involved in detoxification and drug metabolism pathway, were identified in five families (*GSTs, ADH, CYPs, EPHX and AKRs*). Several of these proteins map to or close to RARs. For example, *alcohol dehydrogenase 4* (*ADH4*)*, alcohol dehydrogenase 1C* (*ADH1C*)*, Alcohol dehydrogenase 1A* (*ADH1A*), and *alcohol dehydrogenase 6* (*ADH6*) are on 4q13.3-q35.2, *epoxide hydrolase 2* (*EPHX2*) is on 8p12-p23.2l, and *Aldo-keto reductase family 1 member B10*( *AKR1B10*) *is on* 7q11.2-q35. Previous studies showed tha*t GST, ADH* and *CYP* family members were altered by HBV infections [7,31]. Similarly in this study, we found that the expression of these family members was down-regulated in the HBV(+)/AFB1(+) group and about half of the family members were down regulated in the HBV(+)/AFB1(-) or in the HBV(-)/AFB1(+) group, suggesting both HBV and AFB1 exposure influence expression. Since AFB1 requires detoxification, dysfunction of AFB1 by the phase I and phase II pathways have been implicated in HCC development [32]. These findings suggest that HBV infection might compromise the ability of hepatocytes to detoxify chemical carcinogens by down-regulating the detoxification-related proteins making HBV-infected liver cells more susceptible to carcinogens such as AFB1. This may reflect a synergy between HBV and AFB1.

Up-regulation of *HSPs* has been reported in several cancers (e.g., breast, renal and bladder cancers, various leukemias, as well as HCC.) [33–36] and plays a role in controlling apoptosis and cellular immunity [37–41]. These findings are consistent with our results that found 9 stress-associated proteins (*HSP90AA1, HSP90AB1, GRP78, HSPA1A, GRP75, HSPA8, HSPD1, HSPE1* and *HSPB1*) which were up-regulated in HCC tumors and were involved in the anti-apoptosis and antigen processing and presentation pathways. Over-expression of members of *HSP 70, HSP 90* and *GRPs* gene families in human HBV-associated HCC has been previously described [42] and *HSP 90* has been identified as an essential host factor for HBV replication [43]. These findings suggested *HSPs* may be important for HBV-related HCC. We showed that 9 stress-associated proteins (*HSP90AA1, HSP90AB1 GRP78 HSPA1A GRP75 HSPA8 HSPD1, HSPE1 and HSPB1*) were up-regulated in HBV(+)/AFB1(+), HBV(+)/AFB1(-) and HBV(-)/AFB1(+) groups, suggesting these proteins not only play a role in HBV-related HCC but also in AFB1-related HCC. These factors are primarily involved in anti-apoptotic pathways and antigen-presenting regulation of cell-mediated immunity, and it is possible that changes in the activity of these molecular pathways is result in HCC.

AKR1B10 is highly expressed in solid tumors of several tissues including lung, liver, and pancreas, and thus has received considerable interest as a relevant biomarker for the development of those tumors [44–47]. In addition, increased AKR1B10 expression is associated with smoking in patients with lung cancer and has been proposed to be a potential biomarker for smoking-induced lung cancer [44,46,47]. Currently, it is not clear how AKR1B10 promotes tumorigenesis. It is possible that it may regulate cell proliferation in non-small cell lung cancer as increased AKR1B10 expression is correlated with changes in genes involved in the cell cycles such Ki-67, cyclin E, GalNAcT3, and GnT-V [44,48]. Alternatively, AKR1B10 may promote lung cancer via its enzymatic activity that inhibits the conversion of ß-carotene to retinoic acid and promotes the conversion of highly reactive aldehyde and ketone groups into hydroxyl groups in neoplastic cells resulting in an inhibition of apoptosis [44,49]. In this study, expression of a relatively large number of proteins was altered in the HCC tumors studied. However, we focused on AKR1B10 and evaluated if changes in its expression were associated with HBV or AFB1 exposure for a number of reasons: 1) Our proteomic data suggested that AKR1B10 was expressed at significantly higher levels in patients who were HBV (+) / AFB1 (+) and HBV (-) / AFB1 (+) (9.22 and 5.15, respectively) compared to patients who were HBV (+) / AFB1 (-) or HBV (-) / AFB1 (-) (2.00 and 1.10, respectively); 2) The gene encoding AKR1B10 is located on chromosome 7q33.1, within the high genetic variation region (7q21.1-q35), and regression analysis showed a significant correlation between AKR1B10 expression level and change in its copy number; 3) The tobacco-specific chemical carcinogen NNK, has been shown to be a substrate of AKR1B10 and AKR1B10 is upregulated in smoking-related non-small cell lung cancer. Since AFB1 is a typical chemical carcinogen, we hypothesized that the high expression of AKR1B10 was likely associated with high exposure to AFB1. Moreover, our preliminary data showed that overexpression of AKR1B10 played a role in AFB1-mediated carcinogenesis.

In our study, the expression level of AKR1B10 was higher in the HBV(+)/AFB1(+) and HBV(-)/AFB1(+) groups compared to the HBV(+)/AFB1(-) and HBV(-)/AFB1(-) groups suggesting that AKR1B10 might be involved in AFB1 carcinogen-related hepatocarcinogenesis. Currently, it is not clear if AFB1 has a direct effect by perhaps altering DNA associated with regulatory regions of the AKR1B10 gene, or acts indirectly via one or more molecular pathways to alter AKR1B10 expression. Additional experiments are necessary to investigate this question. 

The major limitation of this study is the small sample size, particularly for the array-based comparative genomic hybridization and isobaric tagging reagent quantitative analysis. It is essential to perform larger studies using samples which have or have not been exposed to HBV or AFB1, in order to further explore the processes involved in HCC. As our analysis relied on tumor tissue, it is not clear which factor or chromosomal aberrations directly influence tumorigenesis or are a result of the cancer. Moreover, this analysis does not elucidate the primary cause of the disease.

## Conclusions

We identified 25 RARs and 133 differentially expressed proteins in HCC tumors from patients in Guangxi, China. Our analysis indicated that loss of 8p12-p23.2 was an independent factor associated with tumor-free survival, loss of 19p13.1-p13.3 may be a genetic characteristic of HCC from Guangxi area, and genetic abnormalities in 4q13.3-q35.2, 13q12.1-q21.2 and 7q11.2-q35 may play a role in both HBV- and AFB1-related HCC carcinogenesis. Our findings also suggest that 1) upregulation of heat-shock proteins and down regulation of metabolic enzymes and detoxification proteins may be common in HBV- and AFB1-related HCC carcinogenesis, and 2) over-expression of AKR1B10 may be involved in AFB1-related HCC carcinogenesis. Taken together, our study provides additional insights into the genetic mechanisms of HBV- and AFB1-related HCC development and demonstrates the complexity of the disease. 

## Supporting Information

Figure S1 **Sequencing results of P53 exon 7 mutation and immunohistochemical staining for AFB1-DNA in HCC: (Left top)P53 gene exon7 mutation (Codon 249 **AGG**>**AGT** ).** (Left bottom) P53 exon 7 mutation (Codon 249 AGG>AGC). (Right top) Positive expression of AFB1-DNA in HCC tissue. (Right bottom) Negative expression of AFB1-DNA in HCC tissue.
(TIF)

Table S1 **The clinical and pathological characteristics of 32 test subjects.**
(DOC)

Table S2 **Association of incidence of recurrently altered regions (RARs) with TNM stage and Edmondson grade in 32 HCC samples.**
(DOC)

Table S3 **The incidence of RARs based on HBV and AFB1 status in 32 HCC samples.**
(DOC)

Table S4 **Up-regulated and down-regulated proteins whose expression levels differed among 4 patient subgroups.**
(DOC)
