# Supplementary figures and images for: Genome-Wide and Differential Proteomic Analysis of Hepatitis B Virus and Aflatoxin B1 Related Hepatocellular Carcinoma in Guangxi, China
Source: PLoS One. 2013 Dec 31;8(12):e83465. doi: 10.1371/journal.pone.0083465 (PMC3877066; doi:10.1371/journal.pone.0083465)

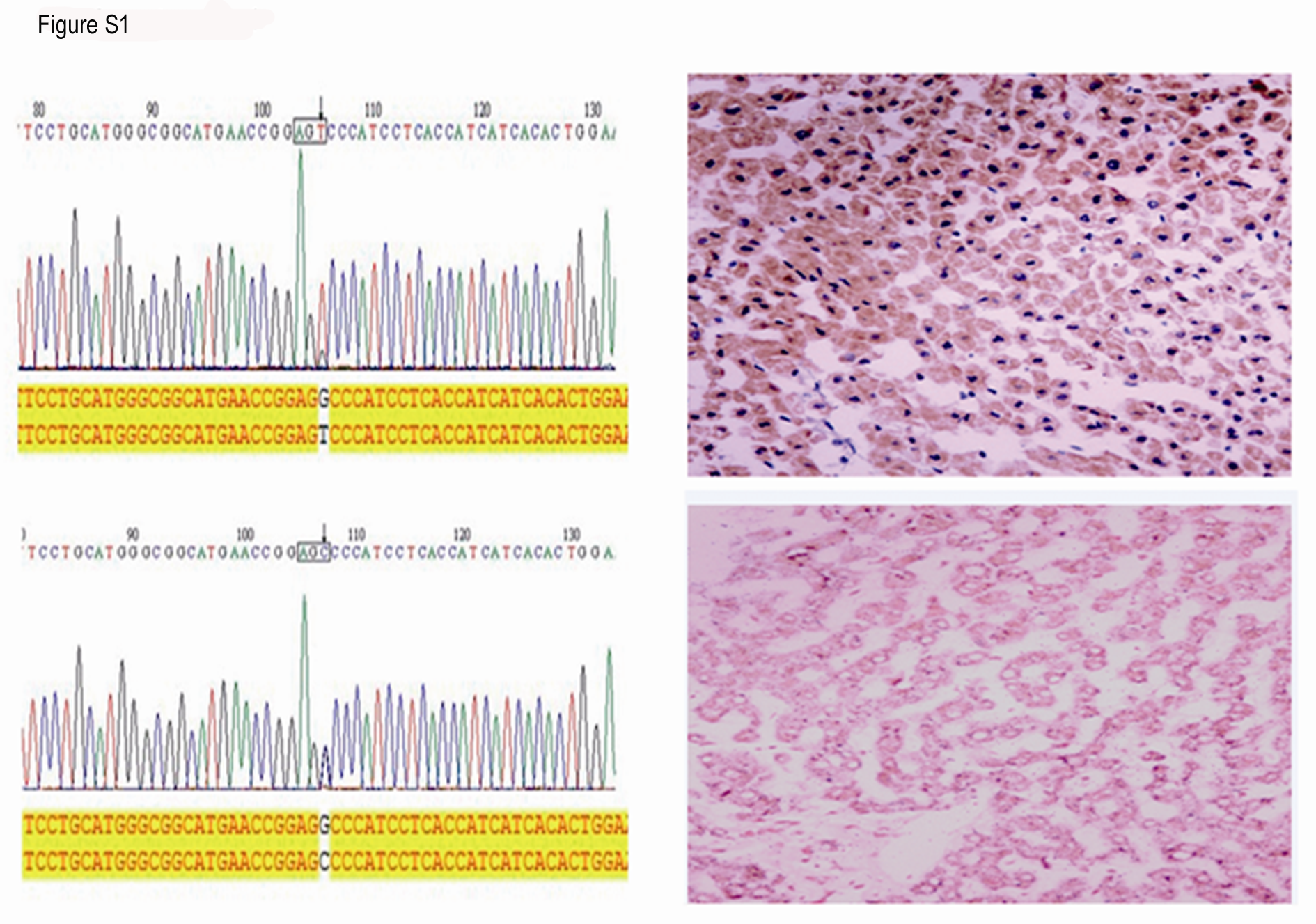

Supplement: Figure S1 — Sequencing results of P53 exon 7 mutation and immunohistochemical staining for AFB1-DNA in HCC: (Left top)P53 gene exon7 mutation (Codon 249 AGG>AGT ). (Left bottom) P53 exon 7 mutation (Codon 249 AGG>AGC). (Right top) Positive expression of AFB1-DNA in HCC tissue. (Right bottom) Negative expression of AFB1-DNA in HCC tissue. (TIF) [file pone.0083465.s001.tif]
