# Supplementary material for: Genome-Wide and Differential Proteomic Analysis of Hepatitis B Virus and Aflatoxin B1 Related Hepatocellular Carcinoma in Guangxi, China
Source: PLoS One. 2013 Dec 31;8(12):e83465. doi: 10.1371/journal.pone.0083465 (PMC3877066; doi:10.1371/journal.pone.0083465)
Supplement: Table S2 — Association of incidence of recurrently altered regions (RARs) with TNM stage and Edmondson grade in 32 HCC samples. (DOC) [file pone.0083465.s003.doc]

| **Table S2. Association of incidence of recurrently altered regions (RARs) with TNM stage and Edmondson grade in 32 HCC samples.** | | | | | | | |
| --- | --- | --- | --- | --- | --- | --- | --- |
| **Chromosome** | **TNM stage** | | **Adjusted P-value^＃^** |  | **Edmondson grade** | | **Adjusted *P*-value^＃^** |
|  | **I~II (n=18)** | **III (n=14)** |  |  | **II (n=17)** | **III (n=15)** |  |
| 1p31.2-p36.2 | 10 (55.6%) | 6 (42.9%) | 0.950 |  | 9 (52.9%) | 7 (46.7%) | >0.999 |
| 1q21.1-q44 | 15 (83.3%) | 5 (35.7%) | 0.083 |  | 10 (58.8%) | 10 (66.7%) | >0.999 |
| 2q23.2-q37.2 | 5 (27.8%) | 6 (42.9%) | >0.999 |  | 4 (23.5%) | 7 (46.7%) | >0.999 |
| 4q13.3-q35.2 | 13 (72.2%) | 9 (64.3%) | >0.999 |  | 13 (76.5%) | 9 (60.0%) | >0.999 |
| 5p13.2-p15.3 | 5 (27.8%) | 6 (42.9%) | >0.999 |  | 6 (35.3%) | 5 (33.3%) | >0.999 |
| 6p12.1-p25.2 | 9 (50.0%) | 4 (28.6%) | 0.903 |  | 6 (35.3%) | 7 (46.7%) | >0.999 |
| 6q14.1-q26 | 6 (33.3%) | 4 (28.6%) | >0.999 |  | 5 (29.4%) | 5 (33.3%) | >0.999 |
| 7q11.2-q35 | 7 (38.9%) | 6 (42.9%) | >0.999 |  | 7 (41.2%) | 6 (40.0%) | >0.999 |
| 8p12-p23.2 | 7 (38.9%) | 13 (92.9%) | 0.038* |  | 7 (41.2%) | 13 (86.7%) | 0.300 |
| 8q11.2-q24.3 | 13 (72.2%) | 9 (64.3%) | >0.999 |  | 11 (64.7%) | 11 (73.3%) | >0.999 |
| 9p21.1-p24.2 | 8 (44.4%) | 4 (28.6%) | 0.981 |  | 5 (29.4%) | 7 (46.7%) | >0.999 |
| 10q21.3-q26.2 | 5 (27.8%) | 7 (50.0%) | 0.989 |  | 7 (41.2%) | 5 (33.3%) | >0.999 |
| 13q12.1-q21.2 | 7 (38.9%) | 6 (42.9%) | >0.999 |  | 8 (47.1%) | 5 (33.3%) | >0.999 |
| 14q21.3-q32.2 | 5 (27.8%) | 8 (57.1%) | 0.621 |  | 7 (41.2%) | 6 (40.0%) | >0.999 |
| 16p12.1-p13.2 | 9 (50.0%) | 5 (35.7%) | 0.942 |  | 6 (35.3%) | 8 (53.3%) | >0.999 |
| 16q12.1-q24.1 | 12 (66.7%) | 9 (64.3%) | >0.999 |  | 8 (47.1%) | 13 (86.7%) | 0.350 |
| 17p12-p13.3 | 13 (72.2%) | 12 (85.7%) | >0.999 |  | 12 (70.6%) | 13 (86.7%) | >0.999 |
| 17q12-q25,2 | 5 (27.8%) | 5 (35.7%) | >0.999 |  | 4 (23.5%) | 6 (40.0%) | >0.999 |
| 18q12.3-q22.3 | 3 (16.7%) | 7 (50.0%) | 0.388 |  | 5 (29.4%) | 5 (33.3%) | >0.999 |
| 19p13.1-p13.3 | 6 (33.3%) | 13 (92.9%) | 0.025* |  | 9 (52.9%) | 10 (66.7%) | >0.999 |
| 19q13.2-q13.4 | 5 (27.8%) | 5 (35.7%) | >0.999 |  | 5 (29.4%) | 5 (33.3%) | >0.999 |
| 21q21.3-q22.2 | 6 (33.3%) | 5 (35.7%) | >0.999 |  | 7 (41.2%) | 4 (26.7%) | >0.999 |
| 22q11.2-q13.2 | 3 (16.7%) | 7 (50.0%) | 0.388 |  | 3 (17.6%) | 7 (46.7%) | >0.999 |
| X | 6 (33.3%) | 5 (35.7%) | >0.999 |  | 7 (41.2%) | 4 (26.7%) | >0.999 |
| Y | 9 (50.0%) | 5 (35.7%) | 0.942 |  | 8 (47.1%) | 6 (40.0%) | >0.999 |

**^＃^**Benjamini and Hochberg procedure was used for control false discovery rate (FDR).

**P*-values ＜ 0.05
