# Supplementary material for: Genome-Wide and Differential Proteomic Analysis of Hepatitis B Virus and Aflatoxin B1 Related Hepatocellular Carcinoma in Guangxi, China
Source: PLoS One. 2013 Dec 31;8(12):e83465. doi: 10.1371/journal.pone.0083465 (PMC3877066; doi:10.1371/journal.pone.0083465)
Supplement: Table S3 — The incidence of RARs based on HBV and AFB1 status in 32 HCC samples. (DOC) [file pone.0083465.s004.doc]

**Table S3. The incidence of RARs based on HBV and AFB1 status in 32 HCC samples**

|  | HBV (-) (n=12) | HBV (+) (n=20) | P-value |  | AFB1 (-) (n=16) | AFB1 (+) (n=16) | P-value |
| --- | --- | --- | --- | --- | --- | --- | --- |
| 1p31.2-p36.2 | 5 (41.7%) | 11 (55.0%) | 0.716 |  | 8 (50.0%) | 8 (50.0%) | >0.999 |
| 1q21.1-q44 | 7 (58.3%) | 13 (65.0%) | 0.724 |  | 12 (75.0%) | 8 (50.0%) | 0.273 |
| 2q23.2-q37.2 | 5 (41.7%) | 6 (30.0%) | 0.703 |  | 5 (31.3%) | 6 (37.5%) | >0.999 |
| 4q13.3-q35.2 | 5 (41.7%) | 17 (85.0%) | 0.018* |  | 9 (56.3%) | 13 (81.3%) | 0.252 |
| 5p13.2-p15.3 | 5 (41.7%) | 6 (30.0%) | 0.703 |  | 8 (50.0%) | 3 (18.8%) | 0.135 |
| 6p12.1-p25.2 | 7 (58.3%) | 6 (30.0%) | 0.150 |  | 6 (37.5%) | 7 (43.8%) | >0.999 |
| 6q14.1-q26 | 4 (33.3%) | 6 (30.0%) | >0.999 |  | 7 (43.8%) | 3 (18.8%) | 0.252 |
| 7q21.1-q35 | 3 (25.0%) | 10 (50.0%) | 0.267 |  | 4 (25.0%) | 9 (56.3%) | 0.149 |
| 8p12-p23.2 | 8 (66.7%) | 12 (60.0%) | >0.999 |  | 9 (56.3%) | 11 (68.8%) | 0.716 |
| 8q11.2-q24.3 | 9 (75.0%) | 13 (65.0%) | 0.703 |  | 9 (56.3%) | 13 (81.3%) | 0.252 |
| 9p21.1-p24.2 | 4 (33.3%) | 8 (40.0%) | >0.999 |  | 5 (31.3%) | 7 (43.8%) | 0.716 |
| 10q21.3-q26.2 | 4 (33.3%) | 8 (40.0%) | >0.999 |  | 4 (25.0%) | 8 (50.0%) | 0.273 |
| 13q12.1-q21.2 | 2 (16.7%) | 11 (55.0%) | 0.062 |  | 4 (25.0%) | 9 (56.3%) | 0.149 |
| 14q21.3-q32.2 | 5 (41.7%) | 8 (40.0%) | >0.999 |  | 6 (37.5%) | 7 (43.8%) | >0.999 |
| 16p12.1-p13.2 | 5 (41.7%) | 9 (45.0%) | >0.999 |  | 8 (50.0%) | 6 (37.5%) | 0.722 |
| 16q12.1-q24.1 | 7 (58.3%) | 14 (70.0%) | 0.703 |  | 12 (75.0%) | 9 (56.3%) | 0.458 |
| 17p12-p13.3 | 8 (66.7%) | 17 (85.0%) | 0.379 |  | 11 (68.8%) | 14 (87.5%) | 0.394 |
| 17q12-q25,2 | 2 (16.7%) | 8 (40.0%) | 0.248 |  | 4 (25.0%) | 6 (37.5%) | 0.704 |
| 18q12.3-q22.3 | 4 (33.3%) | 6 (30.0%) | >0.999 |  | 3 (18.8%) | 7 (43.8%) | 0.252 |
| 19p13.1-p13.3 | 7 (58.3%) | 12 (60.0%) | >0.999 |  | 8 (50.0%) | 11 (68.8%) | 0.473 |
| 19q13.2-q13.4 | 2 (16.7%) | 8 (40.0%) | 0.248 |  | 5 (31.3%) | 5 (31.3%) | >0.999 |
| 21q21.3-q22.2 | 5 (41.7%) | 6 (30.0%) | 0.703 |  | 6 (37.5%) | 5 (31.3%) | >0.999 |
| 22q11.2-q13.2 | 5 (41.7%) | 5 (25.0%) | 0.438 |  | 4 (25.0%) | 6 (37.5%) | 0.704 |
| X | 5 (41.7%) | 6 (30.0%) | 0.703 |  | 6 (37.5%) | 5 (31.3%) | >0.999 |
| Y^※^ | 7 (58.3%) | 7 (35.0%) | 0.277 |  | 6 (37.5%) | 8 (50.0%) | 0.722 |

*Raw p-value < 0.05, but the p-value became non-significant after Benjamini and Hochberg procedure was used.
