# Supplementary material for: Genome-Wide and Differential Proteomic Analysis of Hepatitis B Virus and Aflatoxin B1 Related Hepatocellular Carcinoma in Guangxi, China
Source: PLoS One. 2013 Dec 31;8(12):e83465. doi: 10.1371/journal.pone.0083465 (PMC3877066; doi:10.1371/journal.pone.0083465)
Supplement: Table S4 — Up-regulated and down-regulated proteins whose expression levels differed among 4 patient subgroups. (DOC) [file pone.0083465.s005.doc]

**Table S4. Up-regulated and down-regulated proteins whose expression levels differed among 4 patient subgroups.**

| Accession ID | Protein name (gene name) | (95%) peptides | %COV | Genelocus | iTRAQ ratio | | | |
| --- | --- | --- | --- | --- | --- | --- | --- | --- |
|  |  |  |  |  | HBV(+)/ AFB1(+) (116:113) | HBV(+)/ AFB1(-) (117:113) | HBV(-)/ AFB1(+) (118:113) | HBV(-) /AFB1(-) (119:113) |
|  | **Up-regulated proteins** |  |  |  |  |  |  |  |
| P 17516 | Aldo-keto reductase family 1 member C4 (AKR1C4) | 8 | 47.68 | 10p15.1 | 4.61 | 3.98 | 2.55 | 3.90 |
| P 08238 | Heat shock protein HSP 90-beta (HSP90AB1) | 13 | 42.96 | 6p12^※^ | 6.25 | 3.67 | 7.79 | 2.60 |
| P 07900 | Heat shock protein HSP 90-alpha (HSP90AA1) | 7 | 41.26 | 14q32.3 | 2.08 | 2.73 | 3.37 | 2.33 |
| P 11142 | Heat shock cognate 71 kDa protein (HSPA8) | 11 | 57.13 | 11q24.1 | 3.13 | 4.20 | 4.41 | 3.43 |
| P 04792 | Heat shock protein beta-1 (HSPB1) | 6 | 58.54 | 7q11.23^※^ | 3.72 | 2.33 | 3.46 | 2.01 |
| P31939 | Bifunctional purine biosynthesis protein(ATIC) | 3 | 31.08 | 2q35 | 5.59 | 5.15 | 5.70 | 5.80 |
| Q96QV6 | Histone H2A type 1-A(HIST1H2AA) | 9 | 29.77 | 6p22.2^※^ | 3.16 | 4.13 | 4.47 | 3.16 |
| P10401 | Histone H1.5(HIST1H1B) | 5 | 23.01 | 6p22.1^※^ | 3.80 | 3.90 | 3.70 | 4.00 |
| P 19338 | Nucleolin (NCL) | 4 | 38.45 | 2q37.1 | 3.98 | 3.34 | 4.61 | 2.23 |
| P 62988 | Ubiquitin (RPS27A) | 3 | 75.01 | 2p16 | 3.73 | 2.37 | 3.16 | 2.24 |
| P 23284 | Peptidyl-prolyl cis-trans isomerase B (PPIB) | 2 | 51.39 | 15q21 | 3.56 | 2.85 | 3.19 | 3.40 |
| P14618 | Pyruvate kinase isozymes M1/M2 (PKM2) | 7 | 46.89 | 15q22 | 2.99 | 3.94 | 3.66 | 3.69 |
| P 07237 | Protein disulfide-isomerase (PDIA1) | 15 | 52.38 | 19q22 | 2.96 | 2.42 | 2.10 | 1.97 |
| P04843 | Dolichyl-diphosphooligosaccharide--protein glycosyltransferase | 5 | 39.54 | 1p36.1 | 2.88 | 3.05 | 3.43 | 4.13 |
| Q5VTEO | Putative elongation factor 1-alpha-like 3 (EEF1AL3) | 11 | 56.2 | 9q34.12 | 2.91 | 3.40 | 3.10 | 2.55 |
| P 27797 | Calreticulin (CALR) | 5 | 33.57 | 19p13.3 | 2.75 | 4.28 | 2.67 | 3.95 |
| P 00558 | Phosphoglycerate kinase 1 (PGK1) | 3 | 55.64 | Xq13.3^※^ | 2.55 | 3.25 | 2.72 | 5.29 |
| 43776 | Asparaginyl-tRNA synthetase, cytoplasmic (NARS) | 1 | 12.77 | 18q21.3 | 2.07 | 2.01 | 2.12 | 2.05 |
| Q 00839 | Heterogeneous nuclear ribonucleoprotein U (HNRNPU) | 2 | 41.99 | 1q44^※^ | 2.44 | 2.29 | 2.67 | 2.20 |
| P53999 | Activated RNA polymerase II transcriptional coactivator p15(SUB1) | 2 | 25.98 | 5p13.3^※^ | 2.31 | 2.42 | 2.80 | 2.83 |
| P10155 | 60 kDa SS-A/Ro ribonucleoprotein（TROVE2） | 2 | 20.45 | 1q31^※^ | 2.12 | 2.95 | 2.80 | 2.45 |
| P15104 | Glutamine synthetase(GLUL) | 4 | 25.47 | 1q31^※^ | 4.30 | 2.85 | 2.01 | 2.99 |
| Q 15084 | Protein disulfide-isomerase A6 (PDIA6) | 11 | 51.59 | 2p25.1 | 2.16 | 2.26 | 2.16 | 2.24 |
| P00338 | L-lactate dehydrogenase A (LDHA) | 3 | 47.59 | 11p15.4 | 2.46 | 2.54 | 2.49 | 4.40 |
| P07237 | Protein disulfide-isomerase( P4HB) | 15 | 2.9 | 17q25^※^ | 2.90 | 2.41 | 2.71 | 2.00 |
| 14745 | Ezrin-radixin-moesin-binding phosphoprotein 50 (SLC9A3R1) | 3 | 44.13 | 17q25.1^※^ | 2.11 | 2.63 | 3.16 | 2.01 |
| Q05682 | Caldesmon(CALD1) | 3 | 41.49 | 7q33^※^ | 3.44 | 2.67 | 2.45 | -- |
| P 060218 | Aldo-keto reductase family 1 member B10 (AKR1B10) | 10 | 48.1 | 7q33.1^※^ | 9.22 | 2.00 | 5.15 | -- |
| Q 04828 | Aldo-keto reductase family 1 member C1 (AKR1C1) | 9 | 42.41 | 10p14 | 5.91 | 2.01 | 3.56 | -- |
| P16435 | NADPH--cytochrome P450 reductase(PRO) | 2 | 18.02 | 7q11.28^※^ | 3.19 | 2.10 | 2.23 | -- |
| P 10809 | 60 kDa heat shock protein (HSPD1) | 24 | 63.7 | 2q33.1 | 3.37 | 3.13 | 4.09 | -- |
| P 61604 | 10 kDa heat shock protein (HSPE1) | 3 | 66.67 | 2q33.1 | 2.87 | 2.18 | 2.93 | -- |
| Q 9NS69 | Mitochondrial import receptor subunit TOM22 homolog (TOMM22) | 1 | 27.46 | 22q12 | 2.21 | 2.00 | 2.10 | -- |
| P61981 | 14-3-3 protein gamma (YWHAG) | 2 | 53.04 | 7q11.25^※^ | 2.30 | 2.17 | 2.11 | -- |
| P 06733 | Alpha-enolase (ENO1) | 19 | 56.91 | 1p36.2 | 2.58 | 2.35 | 3.63 | -- |
| P 0C7M2 | Putative heterogeneous nuclear ribonucleoprotein A1-like protein 3 | 3 | 56.56 | 12q13.1 | 2.17 | 2.05 | 2.65 | -- |
| P 62937 | Peptidyl-prolyl cis-trans isomerase A (PPIA) | 9 | 81.72 | 7p13 | 3.40 | 3.69 | 3.63 | -- |
| P 38646 | Stress-70 protein (GRP75) | 16 | 54.05 | 5q31.1 | 2.18 | 2.42 | 2.46 | -- |
| P52758 | Ribonuclease UK114 (HRSP12) | 2 | 70.8 | 8q22^※^ | 2.37 | 2.41 | 2.26 | -- |
| P 11021 | 78 kDa glucose-regulated protein(GRP78) | 14 | 50.76 | 9q33.3 | 2.67 | 2.42 | 2.94 | -- |
| P 07339 | Cathepsin D (CTSD) | 9 | 48.3 | 11p15.5 | 2.10 | 3.53 | -- | -- |
| P30837 | Aldehyde dehydrogenase X, mitochondrial(ALDH1B1) | 4 | 34.43 | 9p11.1 | 3.50 | 2.51 | -- | -- |
| P 13667 | Protein disulfide-isomerase A4 (PDIA4) | 5 | 33.38 | 7q35^※^ | 2.21 | 2.00 | -- | -- |
| P 40926 | Malate dehydrogenase, mitochondrial (MDH2) | 8 | 56.51 | 7q22^※^ | 2.44 | 2.12 | -- | -- |
| Q99471 | Prefoldin subunit 5 (PFDN5) | 1 | 46.75 | 12q12 | 2.83 | -- | 2.07 | 2.35 |
| P 02649 | Apolipoprotein E (APOE) | 5 | 71.61 | 19q13.2 | 2.12 | 2.91 | -- | 2.63 |
| P 08107 | Heat shock 70 kDa protein 1 (HSPA1A) | 10 | 45.09 | 6p21.3^※^ | 2.81 | -- | 2.31 | -- |
| Q05682 | Caldesmon (CALD1) | 3 | 41.49 | 7q33^※^ | 2.44 | -- | 2.42 | -- |
| P23528 | Cofilin-1 (CFL1) | 3 | 42.17 | 11q13 | 2.01 | -- | 2.01 | -- |
| P20700 | Lamin-B1(LMNB1) | 3 | 38.23 | 5q23.2 | -- | 2.44 | 2.18 | 2.48 |
| Q07065 | Cytoskeleton-associated protein 4 (CKAP4) | 3 | 33.89 | 12q23.3 | -- | 2.58 | 2.60 | 2.16 |
| P06899 | Histone H2B type 1-J (HIST1H2BJ) | 14 | 72.22 | 6p22.1^※^ | -- | 2.24 | 2.01 | 2.18 |
| P19971 | Thymidine phosphorylase(TYMP) | 3 | 26,56 | 22q13.3 | -- | 2.78 | -- | 2.75 |
| P62805 | Histone H4 (HIST1H4A ) | 5 | 74.76 | 6p22.1^※^ | -- | 2.10 | -- | -- |
| P61978 | Heterogeneous nuclear ribonucleoprotein K (HNRNPK) | 4 | 41.9 | 9q21.3 | -- | -- | 2.14 | -- |
| P27824 | Calnexin (CANX) | 4 | 22.8 | 5q35 | -- | -- | 2.31 | -- |
| P55072 | Transitional endoplasmic reticulum ATPase (VCP) | 3 | 41.81 | 9p13.3 | -- | -- | 2.67 | 2.14 |
| P68363 | Tubulin alpha-1B chain (TUBA1B) | 10 | 44.35 | 12q13.1 | -- | -- | 2.63 | -- |
| P10599 | Thioredoxin (TXN) | 3 | 58.1 | 9q31 | -- | -- | 2.33 | -- |
|  | **Down-regulated proteins** |  |  |  |  |  |  |  |
| P 08319 | Alcohol dehydrogenase 4 (ADH4) | 7 | 60 | 4q22^※^ | 0.12 | 0.19 | 0.21 | 0.40 |
| Q03154 | Aminoacylase-1 (ACY1) | 7 | 35.29 | 3p21.1 | 0.34 | 0.38 | 0.23 | 0.32 |
| Q9NY33 | Dipeptidyl-peptidase 3 (DPP3) | 3 | 27 | 11q12 | 0.07 | 0.06 | 0.08 | 0.06 |
| O43175 | D-3-phosphoglycerate dehydrogenase(PHGDH) | 4 | 31.89 | 1p12 | 0.25 | 0.10 | 0.03 | 0.21 |
| P05089 | Arginase-1 (ARG1) | 5 | 56.21 | 6q23^※^ | 0.27 | 0.44 | 0.36 | 0.36 |
| Q9BWD1 | Acetyl-CoA acetyltransferase, cytosolic(ACAT2) | 5 | 34.51 | 6q25.3^※^ | 0.47 | 0.38 | 0.44 | 0.40 |
| P01876 | Ig alpha-1 chain C region (IGHA1) | 3 | 42.21 | 14q32.3^※^ | 0.46 | 0.38 | 0.30 | 0.48 |
| P36776 | Lon protease homolog, mitochondrial(LONP1) | 2 | 22.31 | 19p13.2^※^ | 0.38 | 0.38 | 0.47 | 0.46 |
| P36776 | Lon protease homolog, mitochondrial(LONP2) | 1 | 22.31 | 16q12.1^※^ | 0.38 | 0.38 | 0.48 | 0.46 |
| P02765 | Alpha-2-HS-glycoprotein(AHSG) | 2 | 17.71 | 3q27 | 0.28 | 0.22 | 0.32 | 0.15 |
| P36269 | Gamma-glutamyltransferase 5(GGT5) | 1 | 7.58 | 22q11.2^※^ | 0.23 | 0.36 | 0.14 | 0.32 |
| Q3LXA3 | Dihydroxyacetone kinase(DAK) | 8 | 48 | 11q12.2 | 0.43 | 0.28 | 0.04 | 0.07 |
| Q16822 | Phosphoenolpyruvate carboxykinase [GTP], mitochondrial (PCK2) | 13 | 51.41 | 14q11.2^※^ | 0.34 | 0.37 | 0.19 | 0.45 |
| P 05062 | Fructose-bisphosphate aldolase B (ALDOB) | 26 | 56.59 | 9q21.3 | 0.37 | 0.27 | 0.03 | 0.28 |
| Q00796 | Sorbitol dehydrogenase (SORD) | 5 | 47.34 | 15q15.3 | 0.37 | 0.20 | 0.22 | 0.16 |
| Q16851 | UTP--glucose-1-phosphate uridylyltransferase (UGP2) | 3 | 45.47 | 2p14 | 0.39 | 0.47 | 0.12 | 0.24 |
| P 36871 | Phosphoglucomutase-1 (PGM1) | 8 | 43.95 | 1p31^※^ | 0.41 | 0.29 | 0.41 | 0.49 |
| P 08263 | Glutathione S-transferase A1 (GSTA1) | 2 | 43.24 | 6p12.1 | 0.41 | 0.38 | 0.20 | 0.27 |
| P 21333 | Filamin-A (FLNA) | 12 | 29.4 | Xq28 | 0.17 | 0.49 | 0.22 | 0.44 |
| 95954 | Formimidoyltransferase-cyclodeaminase (FTCD) | 10 | 65.43 | 21q22.3^※^ | 0.28 | 0.22 | 0.05 | 0.07 |
| P 00966 | Argininosuccinate synthase (ASS1) | 3 | 34.47 | 9q34.1 | 0.30 | 0.25 | 0.08 | 0.18 |
| 75891 | 10-formyltetrahydrofolate dehydrogenase (ALDH1L1) | 3 | 30.93 | 3q21.3 | 0.46 | 0.25 | 0.27 | 0.39 |
| P 00505 | Aspartate aminotransferase, mitochondrial (GOT2) | 4 | 50 | 16q21^※^ | 0.45 | 0.45 | 0.33 | 0.39 |
| P23141 | Liver carboxylesterase 1( CES1) | 11 | 50.44 | 16q22.2^※^ | 0.45 | 0.39 | 0.46 | 0.45 |
| P 05091 | Aldehyde dehydrogenase, mitochondrial (ALDH2) | 11 | 59.38 | 12q24.2 | 0.49 | 0.49 | 0.15 | 0.46 |
| P06737 | Glycogen phosphorylase, liver form (PYGL) | 1 | 24.44 | 14q21^※^ | 0.45 | 0.29 | 0.32 | 0.20 |
| P 00326 | Alcohol dehydrogenase 1C (ADH1C) | 4 | 38.13 | 4q23^※^ | 0.05 | 0.24 | 0.09 | 0.16 |
| P 21810 | Biglycan (PGS1) | 3 | 26.63 | 17q25.3 | 0.18 | 0.47 | 0.12 | 0.39 |
| Q16836 | Hydroxyacyl-coenzyme A dehydrogenase, mitochondrial(HADH) | 2 | 33.76 | 4q22^※^ | 0.42 | 0.45 | 0.25 | 0.38 |
| Q9Y2P5 | Bile acyl-CoA synthetase (SLC27A5) | 2 | 15.07 | 19q13.4^※^ | 0.38 | 0.21 | 0.20 | 0.25 |
| P 33121 | Long-chain-fatty-acid--CoA ligase 1 (ACSL1) | 5 | 36.82 | 4q35^※^ | 0.20 | 0.15 | 0.10 | 0.15 |
| P50225 | Sulfotransferase 1A1(SULT1A1) | 2 | 36.61 | 16p21.2^※^ | 0.39 | 0.46 | 0.47 | 0.35 |
| P 80404 | 4-aminobutyrate aminotransferase, mitochondrial (ABAT) | 3 | 30.6 | 16p13.2^※^ | 0.43 | 0.24 | 0.07 | 0.29 |
| P02452 | Collagen alpha-1(I) chain (COL1A1) | 3 | 50.82 | 17q21.3 | 0.23 | 0.23 | 0.28 | 0.36 |
| P43488 | Aflatoxin B1 aldehyde reductase member 2(AKR7A2) | 3 | 34.82 | 1p36.1^※^ | 0.11 | 0.50 | 0.43 | 0.47 |
| Q9UBQ7 | Glyoxylate reductase/hydroxypyruvate reductase(GRHPR) | 3 | 24.7 | 9q12 | 0.47 | 0.17 | 0.30 | 0.38 |
| P21810 | Biglycan (BGN) | 3 | 15.22 | Xq28^※^ | 0.18 | 0.47 | 0.21 | 0.39 |
| P07327 | Alcohol dehydrogenase 1A(ADH1A) | 4 | 32 | 4q23^※^ | 0.18 | 0.22 | 0.16 | 0.34 |
| P69892 | Hemoglobin subunit gamma-2 (HBG2) | 5 | 48.33 | 11p15.5 | 0.37 | 0.16 | 0.21 | 0.33 |
| Q93088 | Betaine--homocysteine S-methyltransferase 1 (BHMT) | 8 | 49.75 | 5q13.3 | 0.33 | 0.17 | 0.16 | 0.12 |
| P21964 | Catechol O-methyltransferase(COMT) | 1 | 12.92 | 22q11.2^※^ | 0.47 | 0.44 | 0.36 | 0.28 |
| P34896 | Serine hydroxymethyltransferase, cytosolic(SHMT1) | 2 | 18.43 | 17p12^※^ | 0.19 | 0.24 | 0.45 | 0.45 |
| P30039 | Phenazine biosynthesis-like domain-containing protein(PBLD) | 2 | 27.78 | 10q21.3^※^ | 0.37 | 0.27 | 0.17 | 0.45 |
| P30084 | Enoyl-CoA hydratase, mitochondrial(ECHS1) | 6 | 23.79 | 10q26.2^※^ | 0.19 | 0.50 | 0.10 | 0.32 |
| Q14749 | Glycine N-methyltransferase (GNMT) | 2 | 34.92 | 16p12^※^ | 0.36 | 0.42 | 0.44 | 0.45 |
| P69905 | Hemoglobin subunit alpha (HBA1) | 18 | 56.34 | 16p13.3^※^ | 0.21 | 0.09 | 0.11 | 0.43 |
| P 11586 | C-1-tetrahydrofolate synthase, cytoplasmic (MTHFD1) | 8 | 40 | 14q24^※^ | 0.33 | 0.36 | 0.22 | -- |
| P 68871 | Hemoglobin subunit beta (HBB) | 57 | 89.8 | 11p15.5 | 0.42 | 0.38 | 0.36 | -- |
| P23786 | Carnitine O-palmitoyltransferase 2, mitochondrial（CPT2） | 2 | 27.66 | 1p32^※^ | 0.44 | 0.48 | 0.43 | -- |
| P18206 | Vinculin(VCL) | 4 | 36.07 | 10q22.2^※^ | 0.40 | 0.41 | 0.39 | -- |
| P35580 | Myosin-10 (MYH10) | 6 | 31.73 | 17p13^※^ | 0.34 | 0.43 | 0.38 | -- |
| Q9Y2S2 | Lambda-crystallin homolog (CRYL1) | 3 | 21.63 | 13q12.1^※^ | 0.35 | 0.42 | 0.39 | -- |
| P 05181 | Cytochrome P450 2E1 (CYP2E1) | 2 | 24.34 | 10q24.3^※^ | 0.44 | 0.33 | 0.26 | -- |
| P07099 | Epoxide hydrolase 1 (EPHX1) | 3 | 33.19 | 1q42.1 | 0.33 | 0.47 | 0.44 | -- |
| Q16696 | Cytochrome P450 2A13 (CYP2A13) | 2 | 14.37 | 19q13.2^※^ | 0.30 | 0.44 | 0.16 | -- |
| P28332 | Alcohol dehydrogenase 6 (ADH6) | 1 | 9.23 | 4q23^※^ | 0.46 | 0.49 | 0.44 | -- |
| P 21980 | Protein-glutamine gamma-glutamyltransferase 2 (TGM2) | 3 | 38.14 | 20q12 | 0.46 | -- | 0.22 | 0.11 |
| P 42765 | 3-ketoacyl-CoA thiolase, mitochondrial (ACAA2) | 11 | 47.86 | 18q21.1 | 0.46 | -- | 0.11 | 0.20 |
| P00480 | Ornithine carbamoyltransferase, mitochondrial (OTC) | 4 | 22.88 | Xp21.1 | 0.49 | -- | 0.33 | 0.42 |
| P 02671 | Fibrinogen alpha chain (FGA) | 9 | 28.98 | 4q28^※^ | 0.18 | -- | 0.43 | 0.36 |
| P 08670 | Vimentin (VIM) | 26 | 69.31 | 10p13 | 0.12 | -- | 0.42 | -- |
| P29401 | Transketolase (TKT) | 5 | 23.27 | 3p14.3 | 0.37 | -- | 0.16 | -- |
| P21549 | Serine--pyruvate aminotransferase (AGXT) | 5 | 42.09 | 2q37^※^ | 0.43 | -- | 0.34 | -- |
| P 12111 | Collagen alpha-3(VI) chain (COL6A3) | 7 | 27.7 | 2q37^※^ | 0.23 | -- | 0.34 | -- |
| Q06520 | Bile salt sulfotransferase(SULT2A1) | 3 | 39.65 | 19q13.3^※^ | 0.40 | -- | 0.35 | -- |
| Q9Y2V2 | Calcium-regulated heat stable protein 1(CARHSP1) | 2 | 31.97 | 16p13.2^※^ | 0.40 | -- | 0.45 | -- |
| P00441 | Superoxide dismutase [Cu-Zn] (SOD1) | 3 | 50.65 | 21q22.1^※^ | 0.44 | -- | 0.44 | -- |
| Q7Z4W1 | L-xylulose reductase (DCXR) | 5 | 69.26 | 17q25.3 | -- | 0.34 | 0.31 | 0.40 |
| P34913 | Epoxide hydrolase 2 (EPHX2) | 2 | 28.29 | 8p21.2^※^ | 0.44 | 0.49 | -- | -- |
| Q02318 | Cytochrome P450 27, mitochondrial (CYP27A1) | 2 | 8.28 | 2q33^※^ | 0.12 | 0.48 | -- | -- |
| Q06278 | Aldehyde oxidase(AOX1) | 3 | 26.01 | 2q33^※^ | 0.45 | 0.30 | -- | -- |
| P35579 | Myosin-9 (MYH9) | 19 | 10.36 | 22q13.1^※^ | 0.46 | -- | -- | -- |
| Q92734 | Protein TFG (TFG) | 3 | 17.25 | 2q12.2^※^ | 0.45 | -- | -- | -- |
| P 02675 | Fibrinogen beta chain (FGB) | 2 | 27.9 | 4q28^※^ | 0.27 | -- | -- | -- |

**^※^** The differentially expressed proteins mapped within recurrently altered regions (RARs）
